# From behavioral to structural correlates: a multi-phase analysis of global reported mpox

**DOI:** 10.1186/s12916-026-05068-4

**Published:** 2026-07-21

**Authors:** Li Shen, Mengna Wei, Yu Chen, Rui Li, Zhenfan Xu, Yuetong Chen, Yaqin Su, Mengyan Ye, Zhenhua Lu, Jiayi Li, Jinzhan Chen, Junze Du, Yijie Yin, Ying Liu, Peng Li, Liqun Fang, Xiaojie Yuan, Wei Liu, Kun Liu

**Affiliations:** 1 School of Remote Sensing and Information Engineering, Wuhan University, Wuhan, 430079 China; 2 Department of Epidemiology, The Ministry of Education Key Lab of Hazard Assessment and Control in Special Operational Environment, The Shaanxi Provincial Key Laboratory of Environmental Health Hazard Assessment and Protection, The Shaanxi Provincial Key Laboratory of Free Radical Biology and Medicine, School of Public Health, The Fourth Military Medical University, Xi’an, 710032 China; 3 State Key Laboratory of Pathogen and Biosecurity, Academy of Military Medical Science, Beijing, 100071 China

**Keywords:** Mpox, Epidemiological phases, Macro-level correlates, Structural vulnerability

## Abstract

**Background:**

Mpox epidemiology has expanded from endemic zoonotic spillover to sustained human-to-human transmission. While behavioral risk factors have been well recognized, how macro-level correlates of mpox burden vary across epidemic phases remains insufficiently quantified, limiting targeted public health responses.

**Methods:**

Using World Health Organization (WHO) global daily mpox surveillance data from January 1, 2022 to April 30, 2025, we calculated country-period reported mpox incidence and developed a phase-sensitive analytical framework integrating multi-domain macro-level variables across distinct epidemic phases. Five ensemble machine learning models (eXtreme Gradient Boosting, Gradient Boosting Decision Tree, Light Gradient Boosting Machine, Random Forest, Categorical Boosting) were optimized, and their outputs were interpreted using SHapley Additive exPlanations (SHAP) values to identify the changing correlates of mpox burden across phases.

**Results:**

Reported mpox incidence exhibited four temporal-epidemiological phases, comprising localized sporadic emergence, accelerated global spread, sustained dissemination, and region-specific resurgence. SHAP-based interpretation revealed a phase-dependent shift in model-associated correlates, from behavioral factors in earlier phases, such as close-contact networks and population mobility, to later structural vulnerability indicators related to socioeconomic disparities and health-system capacity. The LGBT+ (lesbian, gay, bisexual, transgender, and other sexual and gender minorities) Legal Index was the strongest overall predictor, accounting for 13.5% of the overall SHAP importance. Its association with reported mpox incidence was context-dependent, with positive contributions occurring in legally inclusive settings and negative contributions observed in restrictive settings, a pattern consistent with differential case ascertainment and reporting visibility.

**Conclusions:**

Our findings highlight a transition from behavioral to structural indicators in global mpox burden, uncovering phase-specific and geographic heterogeneity. These results support phase-tailored interventions that combine early targeted prevention with later health-system strengthening, equitable surveillance, community support, and stigma reduction.

**Supplementary Information:**

The online version contains supplementary material available at https://doi.org/10.1186/s12916-026-05068-4.

## Background

Mpox is a zoonotic, smallpox-like disease caused by mpox virus (MPXV), a member of the species *Orthopoxvirus monkeypox* [1]. Since its first human documentation in 1970 in the Democratic Republic of the Congo [2], mpox remained endemic in Central and West African rainforest ecosystems for decades [3–5]. However, its epidemiological landscape has undergone a dramatic shift since 2022 [6], with sustained human-to-human transmission becoming prominent during the international outbreak. The current global outbreak began with a case reported in the United Kingdom on May 7, 2022, and rapidly escalated into unprecedented transcontinental transmission [7]. As of April 30, 2025, a total of 138,830 mpox cases had been reported across 133 countries and territories. The World Health Organization (WHO) has twice declared a Public Health Emergency of International Concern (PHEIC) in response to the epidemic [8, 9], underscoring mpox’s transition from a regionally confined African zoonosis to a pressing global health threat [10, 11].

Disease surveillance during the 2022 international outbreak associated with Clade IIb identified a marked concentration of early reported cases among men who have sex with men (MSM) [12–14], with close-contact sexual networks and multiple sexual partnerships contributing to rapid early spread. Globalization has intensified international mobility [15, 16] and expanded interconnected travel and trade networks [17, 18], facilitating cross-border dissemination and extending human-to-human transmission chains [19]. Public health interventions, including targeted vaccination [20], contact tracing [21], and community health education [22], have demonstrated partial effectiveness in curbing mpox transmission.

However, the prevailing literature has largely emphasized behavioral factors, particularly individual- or group-level practices proximal to exposure risk, such as sexual contact networks and human mobility. This paradigm has left the role of macro-level structural conditions insufficiently examined, including socioeconomic inequality, health-system capacity, legal and institutional environments, and infrastructure, all of which shape population vulnerability and access to care. While behavior-focused studies have clarified early outbreak clustering, they cannot fully explain why mpox burden has varied so markedly across epidemiological phases and regions [23]. These patterns may reflect phase-varying contributions from behavioral factors and broader structural conditions. To move beyond these limitations, three interrelated questions warrant systematic investigation: (1) What epidemiological phases have characterized global mpox incidence since 2022? (2) How have macro-level correlates of mpox incidence varied across phases and regions? (3) How are sociocultural contexts associated with geographic heterogeneity in mpox burden?

**Fig. 1:**
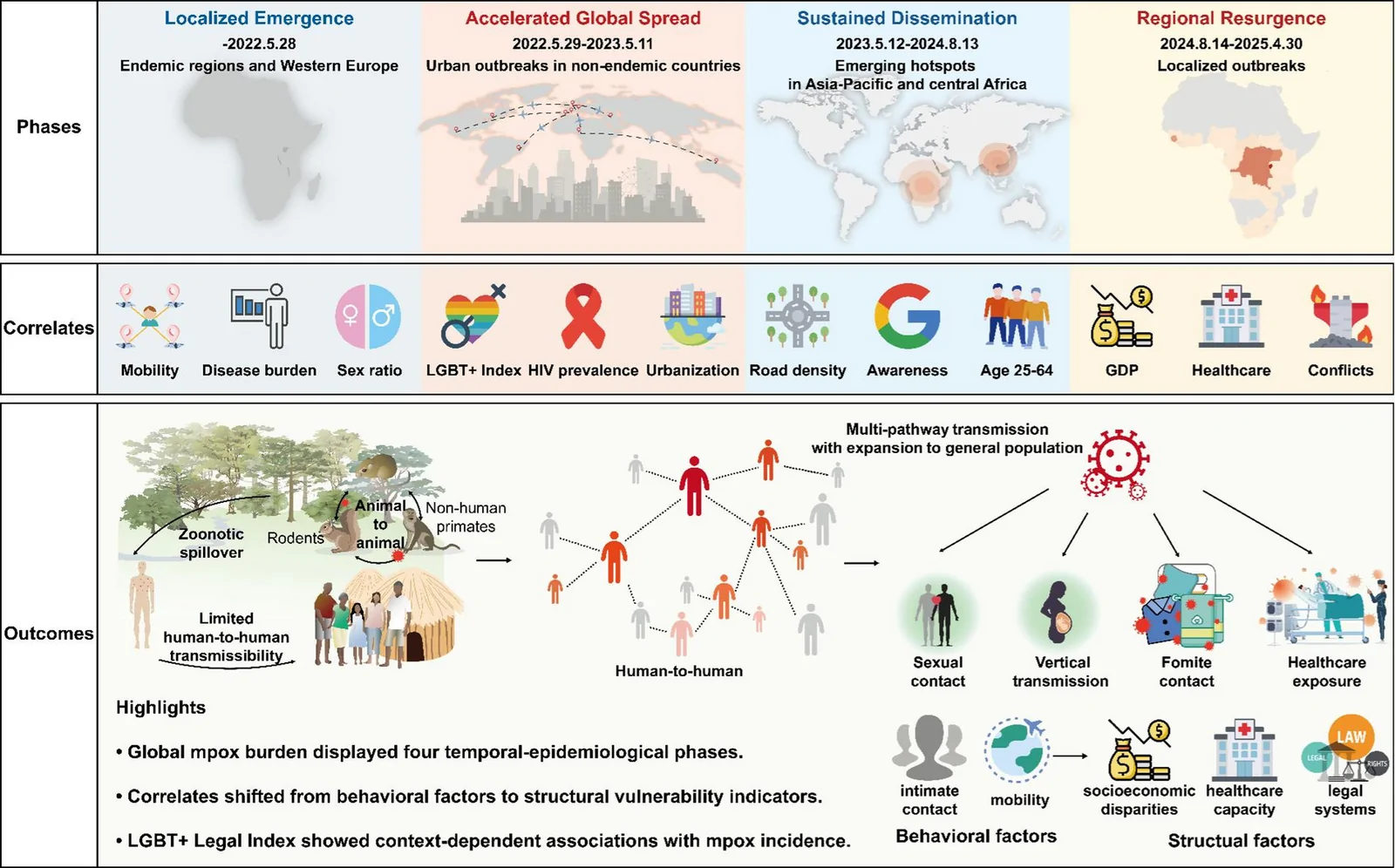
Phase-sensitive theoretical framework for global mpox transmission dynamics

This study proposes a phase-sensitive analytical framework (Fig. 1) to examine changes in macro-level correlates of reported mpox incidence across epidemic phases. By integrating multisource socioeconomic, environmental, healthcare, and mobility data, we implemented an interpretable, phase-stratified modeling approach combining five machine learning algorithms with SHapley Additive exPlanations (SHAP) to quantify the relative importance of candidate correlates across phases. Our work not only advances the understanding of how macro-level factors shape reported mpox burden across distinct epidemiological phases, but also provides actionable evidence for designing context-adaptive public health responses.

## Methods

### Data and preprocessing

Global daily mpox case data from January 1, 2022 to April 30, 2025, were obtained from the WHO surveillance database [24]. Demographic and epidemiological covariates, including gender, age, and transmission mode, were systematically extracted. Based on the phase-sensitive analytical framework, we calculated the mpox incidence for each phase and country, defined as the cumulative reported cases per 100,000 population. To contextualize lineage heterogeneity, we retrieved available MPXV sequence data from Global Initiative on Sharing All Influenza Data (GISAID) and summarized country-level lineage composition according to the epidemic phases predefined in this study. These genomic data were used solely for descriptive lineage contextualization and were not linked to individual WHO-reported cases, used to assign clade status to WHO surveillance records, or used to estimate clade-specific incidence.

Drawing upon previous research on influential factors associated with mpox [25], 41 potential variables were selected and further categorized into seven primary domains: socio-demographic, socioeconomic, socio-cultural, healthcare, disease burden, transportation, and environmental sanitation (Additional file 1: Table S1) [26–44]. These variables covered a range of static factors, such as Gross Domestic Product per capita (GDP) and the International Health Regulations (IHR), and dynamic factors, such as coronavirus disease 2019 (COVID-19) cases and Google Trends (Additional file 2: Figs. S1–S2). Several variables exhibited missing values owing to the multi-country design (Additional file 1: Table S1), which we addressed via a K-Nearest Neighbors imputation algorithm (k = 5) (Additional file 2: Figs. S3–S6). All variables were then standardized for consistency and comparability across the dataset.

### Feature selection using RFECV

To identify the most influential factors associated with reported mpox incidence, we employed Recursive Feature Elimination with Cross-Validation (RFECV) to select the optimal feature subsets from the 41 candidate variables.

RFECV is a feature selection method that iteratively removes less informative variables and identifies an optimal subset based on cross-validated model performance, thereby reducing redundancy and improving predictive efficiency (Additional file 3: Text S1) [45–47]. The selected features varied across different study phases (Additional file 1: Tables S2–S6, Additional file 2: Fig. S7). According to the phase-sensitive analytical framework, candidate correlates were evaluated separately by phase to characterize temporal changes in their model contributions across the mpox epidemic phases.

To further address multicollinearity among the selected features, we conducted pairwise Pearson correlation analysis, flagging feature pairs with strong linear relationships (|r| > 0.8 and *p* < 0.05). For each correlated pair, the variable with the lower model-derived importance score was removed (Additional file 2: Figs. S8–S9). This two-stage selection strategy ensured final feature sets were highly informative and statistically non-redundant, improving model interpretability and stability.

### Machine learning models

Since the associations between features and reported mpox incidence might vary across epidemic phases, using the same single model may not achieve optimal performance for all phases. Therefore, we utilized five models and compared their performances to ensure the robustness and avoid bias, which is also a common practice in relevant machine learning studies [48, 49]. The five machine learning models included eXtreme Gradient Boosting (XGBoost), Gradient Boosting Decision Tree (GBDT), Light Gradient Boosting Machine (LightGBM), Random Forest, and Categorical Boosting (CatBoost). These models were implemented to explore the relationships between mpox incidence and candidate variables and were selected for their ability to capture nonlinear associations, handle heterogeneous feature types, and generate robust predictions (Additional file 3: Text S2) [50–54].

Hyperparameter optimization was further performed using the automated Optuna framework, systematically searching for optimal parameters for each model (Additional file 1: Table S7). The dataset was split 8:2 into training and testing sets to ensure model generalization. Model performance was evaluated using Root Mean Squared Error, Mean Absolute Error, and the coefficient of determination (R²). The final tuned parameters and corresponding performance metrics for each model across different epidemic phases are presented in Additional file 1: Table S8. To interpret model outputs, we applied SHAP to decompose model predictions into individual feature contributions and identify variables associated with predicted reported incidence (Additional file 3: Text S3) [55–59].

## Results

### Epidemiological trends and population susceptibility of mpox

**Fig. 2:**
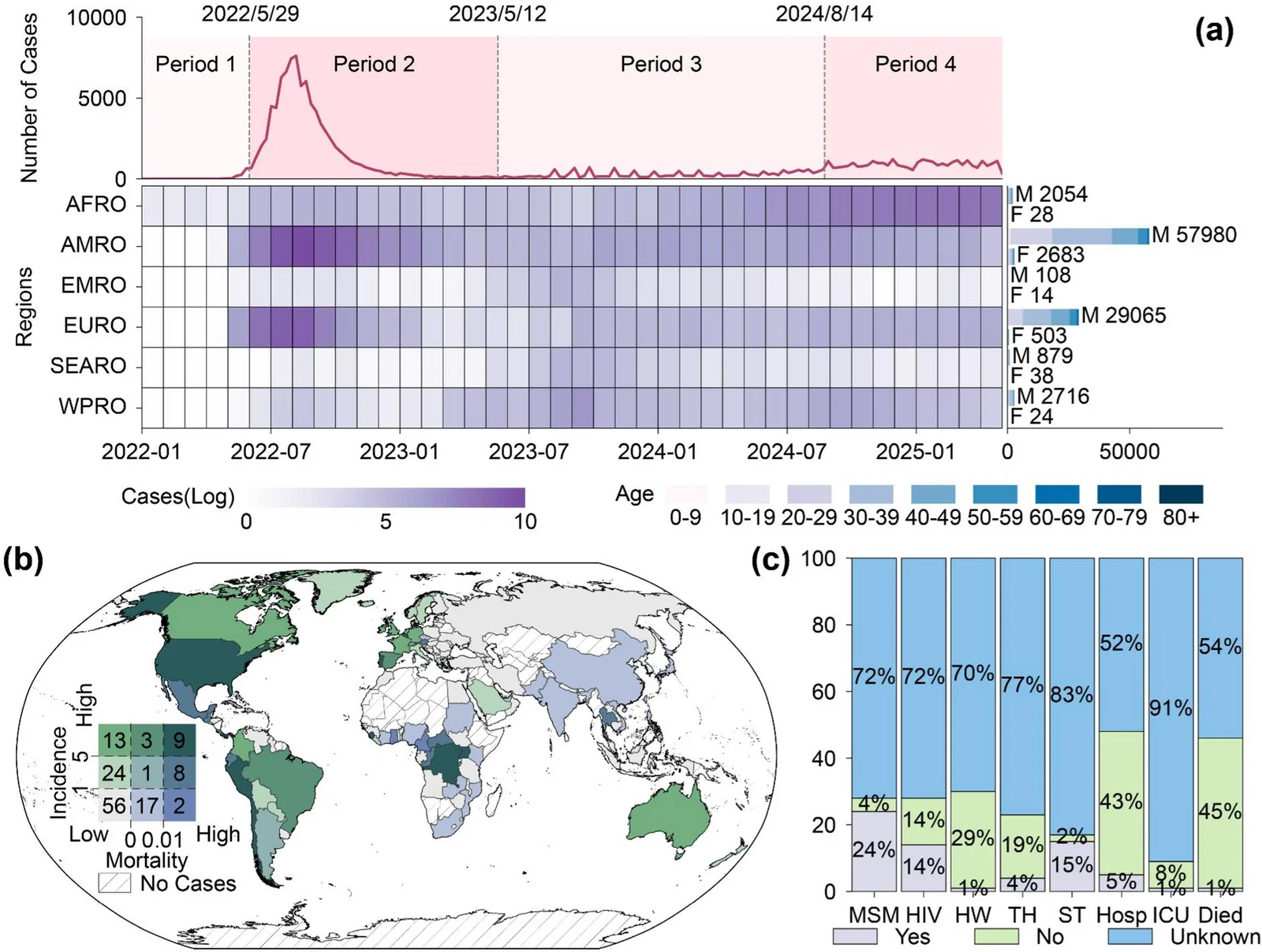
Epidemiological characteristics of mpox from January 1, 2022, to April 30, 2025, globally and across WHO regions. **a** The upper panel shows weekly global mpox cases, while the lower panel presents monthly case distribution heatmaps for WHO regions, with deeper purple indicating a higher number of cases (log-transformed). The stacked bar chart on the right displays the distribution of mpox cases across regions by gender and age group. **b** A dual-color map illustrating mpox incidence and mortality rates globally. **c** Demographic information of mpox cases, MSM - Men who have sex with men, HIV - Persons living with HIV, HW - Health worker, TH - Travel History, SW - Sexual Transmission, Hosp - Hospitalized

Global reported mpox incidence was characterized by four temporal-epidemiological phases from January 2022 to April 2025, as outlined in our phase-sensitive framework (Additional file 1: Table S9; Fig. 2a). During Phase 4, reported cases were concentrated in the African Region, which accounted for 77% (25,667/33,329) of the total cases in that phase. The epidemic predominantly affected young and middle-aged adult males, who represented 96.6% of cases, with the 20–39 age group bearing the majority (68.0%) of the global burden (Additional file 1: Table S10). Available GISAID genomes showed substantial lineage heterogeneity across phases. Among these genomes, Clade IIb predominated in Periods 1 and 2, accounting for 96.3% (287/298) and 97.6% (8,985/9,208), respectively. In Period 3, Clade I accounted for 25.0% of the available GISAID genomes. In Period 4, Clade I accounted for 67.7% (2,506/3,699) of the available GISAID genomes overall and 93.1% of those from the African Region, with Clade Ib accounting for 49.0% (Additional file 1: Table S11; Additional file 2: Fig. S10).

While cases in Europe and the Americas peaked sharply among adults aged 30–39, the African Region exhibited a significant pediatric skew (Fig. 2b), suggesting differential transmission dynamics. The epidemiological profile was characterized by a high concentration of cases among MSM (86.7%), with sexual contact as the primary transmission route (88.4%), alongside a high human immunodeficiency virus (HIV) co-infection rate (51.5%), and recent international travel (16.9%) (Additional file 1: Table S12; Fig. 2c).

### Macro-level correlates of reported mpox incidence throughout the study period

**Fig. 3:**
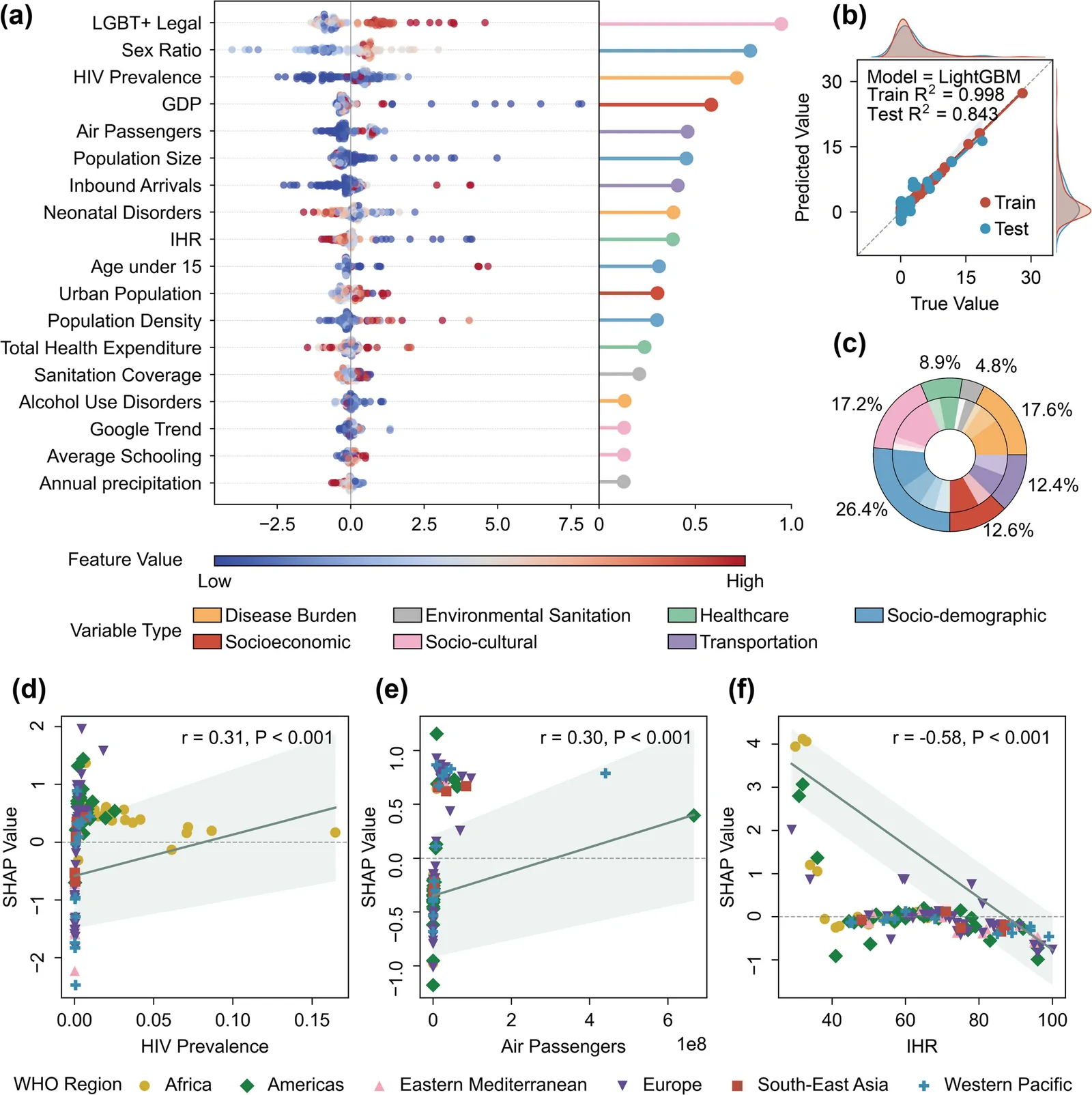
SHAP interpretability analysis of mpox incidence during the study period. **a** Global SHAP summary plot illustrating feature contributions to mpox incidence predictions. Left: scatter plot of individual SHAP values per feature across countries, colored by feature value (red = high, blue = low). Right: lollipop plot showing the mean absolute SHAP value for each feature, indicating its overall importance. **b** Scatter plot of predicted vs. true mpox incidence values for the LightGBM model on both training and test sets. **c** Pie chart of grouped feature importance by variable category, based on aggregated Mean |SHAP| values. **d** SHAP dependence plot for HIV prevalence. **e** SHAP dependence plot for air passenger volume. **f** SHAP dependence plot for International Health Regulations (IHR) core capacity scores

A LightGBM-based machine learning model demonstrated high predictive accuracy for reported mpox incidence (Test *R²* = 0.843; Fig. 3b). SHAP analysis provided insights into potential associations between predictor variables and mpox incidence (January 2022 to April 2025).

Globally, the LGBT+ Legal Index was the most influential single variable of model-predicted reported mpox incidence (mean |SHAP| = 0.947; 13.5% contribution; Fig. 3a). Socio-demographic factors exhibited the highest aggregated feature importance (26.4%), followed by disease burden (17.6%) and socio-cultural indicators (17.2%) (Fig. 3c). Higher HIV prevalence was associated with higher predicted incidence in the Americas and Africa (Fig. 3d). Cross-border mobility, represented by air passenger volume, was linked with the increased incidence when its volume exceeded 7 million (Fig. 3e). Besides, there existed an obvious negative association between IHR core capacity scores and mpox incidence (Fig. 3f), which may be affected by more effective containment, surveillance, or vaccine availability in high-IHR settings.

Regional heterogeneity analysis (Additional file 2: Fig. S11) demonstrated socio-demographic (32.4%) and socioeconomic (23.0%) factors accounted for a larger contribution in Africa, whereas disease burden (29.7%) factors performed more prominent in the Eastern Mediterranean region. Other regions exhibited a more balanced distribution of influencing factors, with socio-cultural variables such as the LGBT+ Legal Index among the notable contributors.

### Regional heterogeneity stratified by LGBT+ Legal Index

**Fig. 4:**
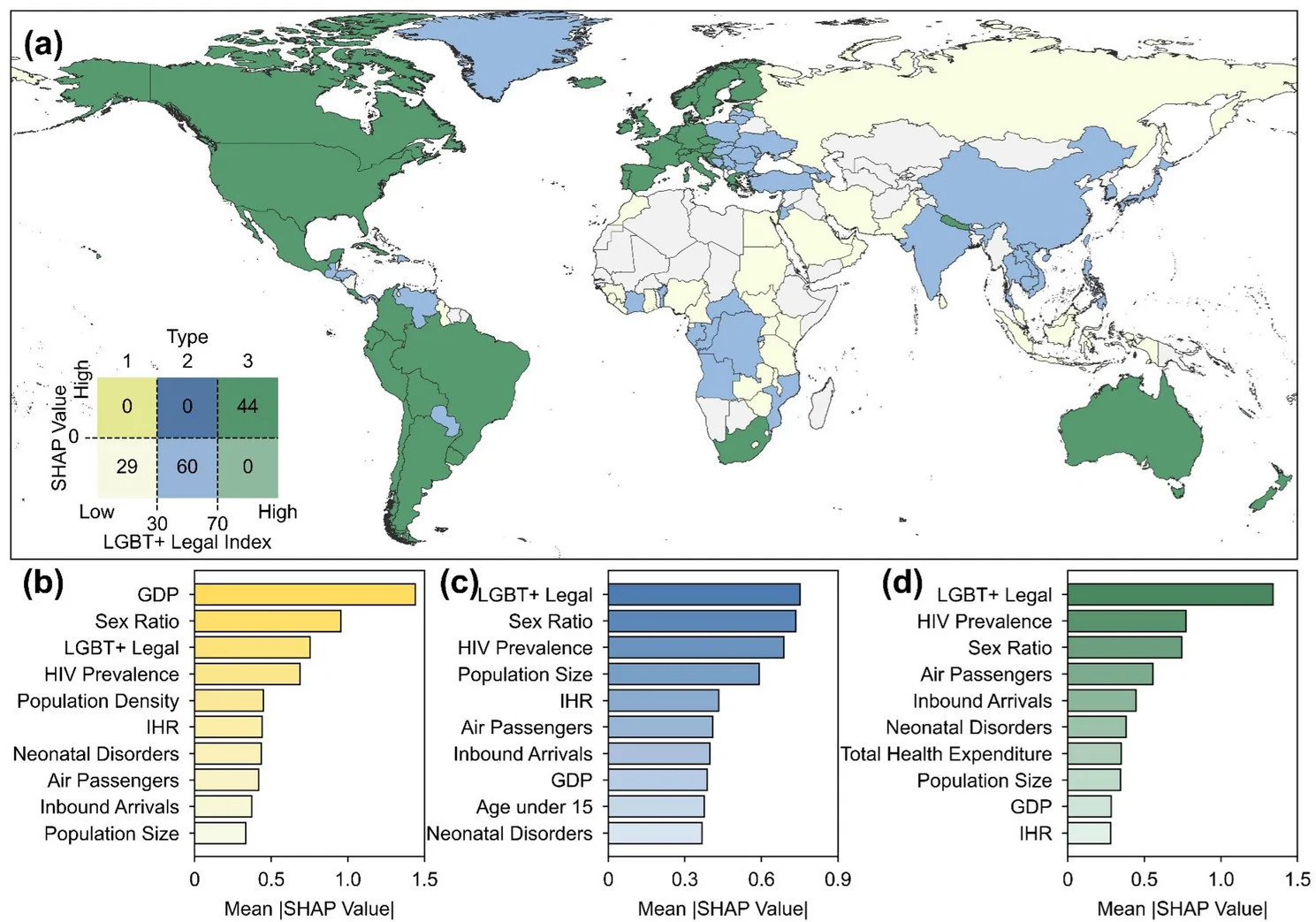
Classification of countries based on the LGBT+ Legal Index and key contributors of mpox incidence in different categories. **a** Countries were categorized into three groups based on the LGBT+ Legal Index: Type 1 (LGBT+ Legal Index ≤ 30, minimal legal protections), Type 2 (30 < LGBT+ Legal Index ≤ 70, moderate legal protections), and Type 3 (LGBT+ Legal Index > 70, strong legal protections). The map illustrates the classification of countries and their corresponding SHAP contribution directions. **b**–**d** The top 10 variables influencing mpox incidence across different LGBT+ Legal Index categories, ranked by SHAP value importance: (**b**) Type 1 countries (LGBT+ Legal Index ≤ 30, negative SHAP values ). **c** Type 2 countries (30 < LGBT+ Legal Index ≤ 70, negative SHAP values). **d** Type 3 countries (LGBT+ Legal Index > 70, positive SHAP values)

The LGBT+ Legal Index, a composite measure of legal protections for LGBT+ individuals, emerged as a key predictor of reported mpox incidence. Given that this country-level variable may partly reflect surveillance visibility and differential case ascertainment rather than direct transmission risk, countries were categorized into three groups based on index scores to explore spatial heterogeneity across legal and sociocultural contexts (Fig. 4a).

#### Type 1 (LGBT+ Legal Index ≤ 30; minimal legal protections)

In this group, the LGBT+ Legal Index showed a negative SHAP contribution to predicted reported incidence (mean |SHAP| = 0.754). GDP per capita (18.5%) and sex ratio were the leading feature contributors (Fig. 4b).

#### Type 2 (30 < LGBT+ Legal Index ≤ 70; moderate legal protections)

A similar negative SHAP contribution was observed for the LGBT+ Legal Index (mean |SHAP| = 0.751). Sociodemographic variables, including sex ratio, population size, and age < 15, collectively contributed 31.0% to the model explanation (Fig. 4c).

#### Type 3 (LGBT+ Legal Index > 70; strong legal protections)

By contrast, the LGBT+ Legal Index contributed positively to predicted reported incidence (mean |SHAP| = 1.340). Sociocultural factors accounted for the largest aggregate contribution (23.6%), driven primarily by the LGBT+ Legal Index, while Air Passengers and Inbound Arrivals also ranked among the top contributors (mean |SHAP| = 0.556 and 0.446, respectively; Fig. 4d).

## Phase-specific correlates of reported mpox incidence

**Fig. 5:**
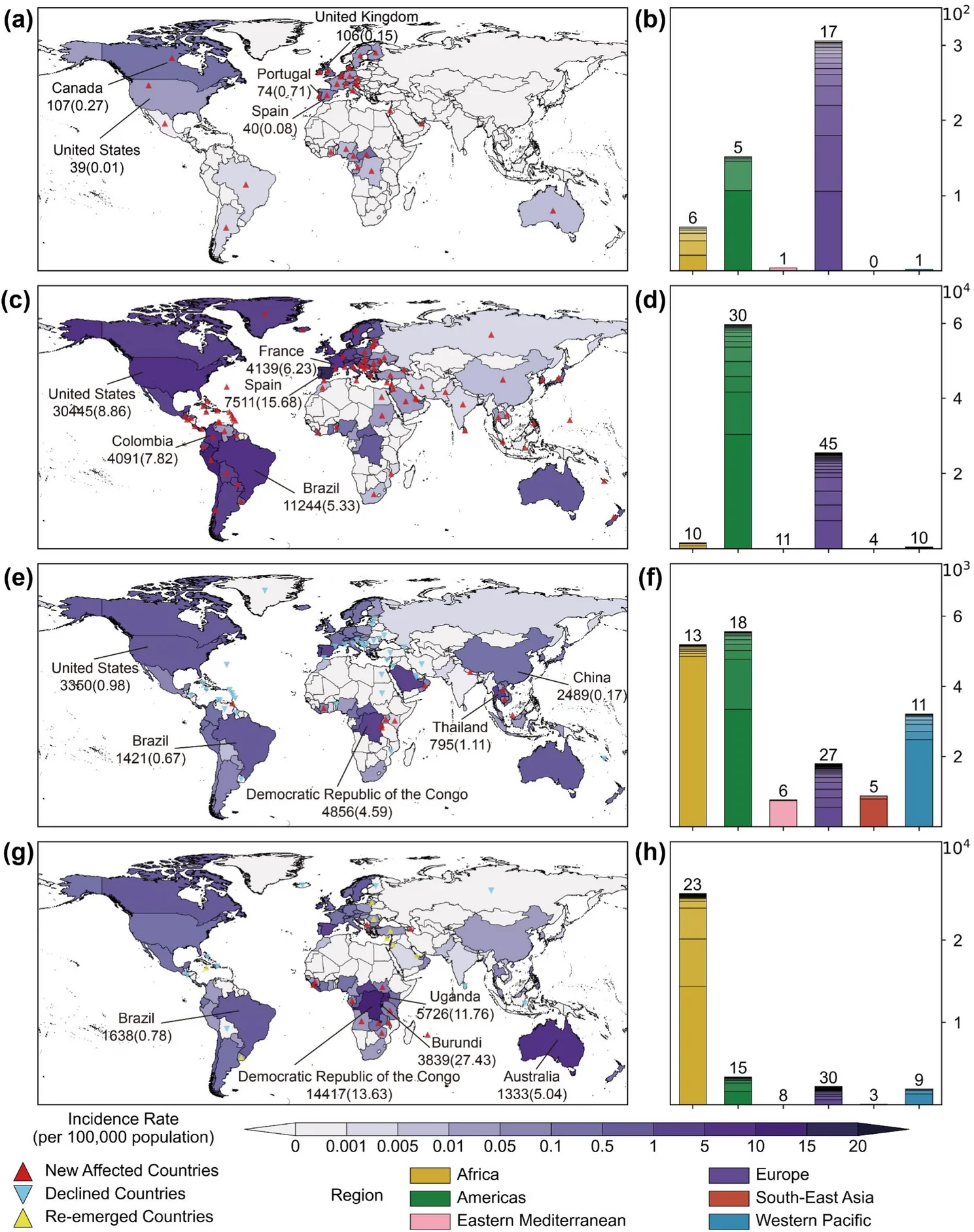
Global mpox incidence and regional distribution across different study periods. **a** Period 1, (**c**) Period 2, (**e**) Period 3, and (**g**) Period 4: Global mpox incidence rates and the top five countries with the highest case counts during each period. **b** Period 1, (**d**) Period 2, (**f**) Period 3, and (**h**) Period 4: Bar charts showing the total number of mpox cases and the number of reporting countries in each region

**Fig. 6:**
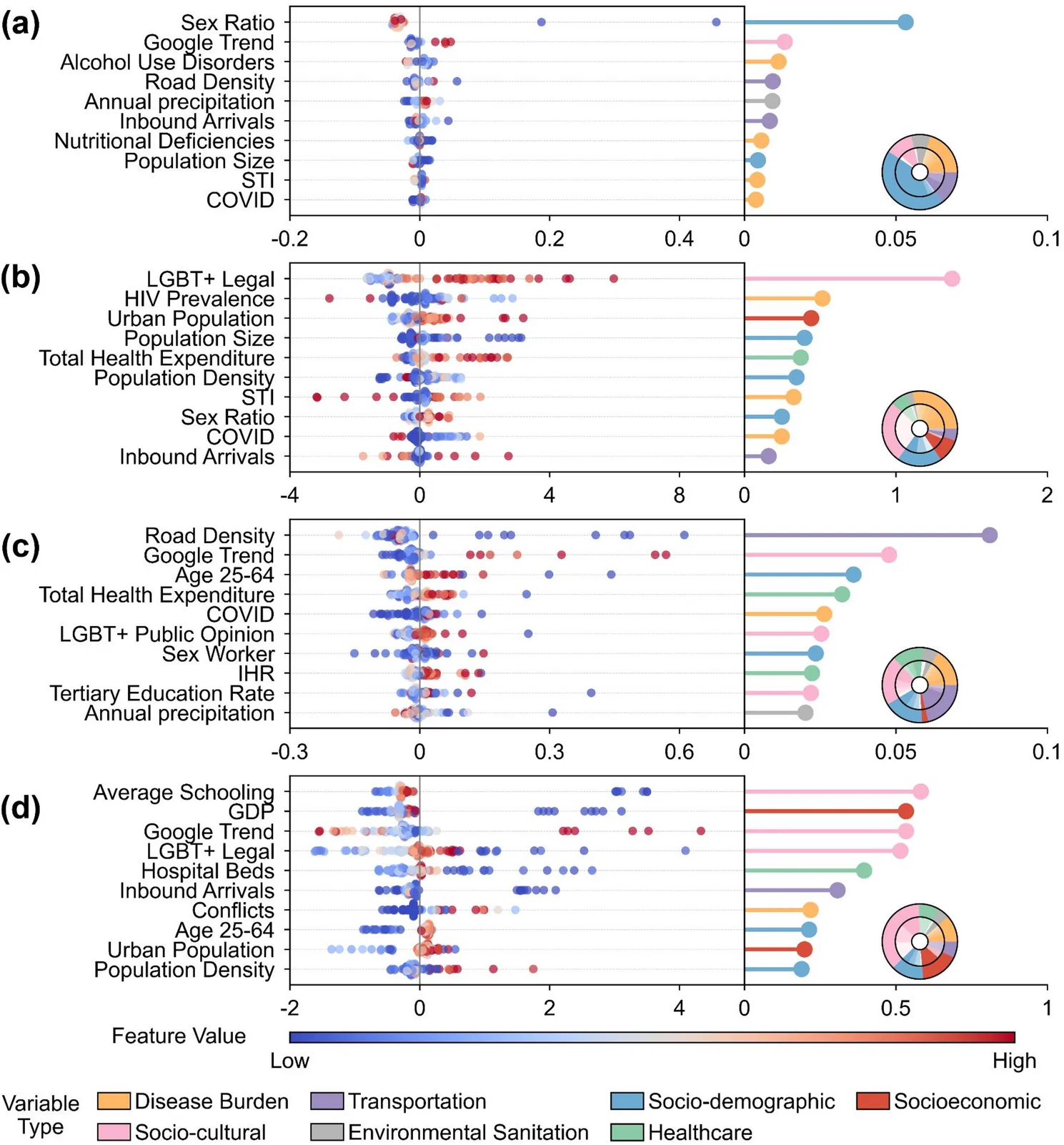
Phase-specific correlates of reported mpox incidence across four epidemic periods. **a** Period 1, (**b**) Period 2, (**c**) Period 3, and (**d**) Period 4. For each period, beeswarm plots (left panels) display feature impacts and distributions for top 10 variables, with red/blue shading denoting high/low feature values. Lollipop charts (right panels) highlight the contributors by mean absolute SHAP value magnitude, color-coded by variable category; inset polar charts decompose these hierarchically, with outer arcs representing category totals and inner segments individual feature proportions

Consistent with the phase-sensitive analytical framework, we examined reported mpox incidence across four distinct phases (Fig. 5) and evaluated the performance of five machine learning models according to the phase-specific data characteristics. Optimal models were selected based on the highest out-of-sample *R²* (Additional file 1: Table S13; Additional file 2: Fig. S12), and SHAP analysis was subsequently used to explore the key variables associated with predicted reported incidence in each phase (Fig. 6).

During the localized sporadic phase (**Period 1**), the global case burden remained low, with only three countries reporting over 50 cases (Fig. 5a), concentrating in Western Europe and North America (Fig. 5b). SHAP analysis revealed low overall feature importance (mean |SHAP|<0.1), with sex ratio as the most influential feature, consistent with sparse observations and high uncertainty in this phase (Fig. 6a).

During the accelerated global spread phase **(Period 2**), the epidemic expanded to Latin America, Central Europe, and parts of Asia, accompanied by a substantial surge in mpox incidence rates. Spain reported the highest incidence rate, and the United States reported more than 30,000 confirmed cases (Fig. 5c-d). SHAP identified LGBT+ Legal Index as the leading contributor to model-predicted reported incidence (mean |SHAP| = 1.370), with higher index scores making positive contributions to predicted incidence. Urban population, inbound travel, and health expenditure were also identified as notable contributors (Fig. 6b).

During the sustained dissemination phase (**Period 3**), several island nations and Central European countries ceased reporting new mpox cases (Fig. 5e), while Central Africa and Southeast Asia emerged as newly affected regions (Fig. 5f). Road density was positively associated with mpox incidence in countries such as the Democratic Republic of the Congo (SHAP = 0.611) and Saudi Arabia (SHAP = 0.485). Demographic variables, including Age 25–64 and Sex Worker, also emerged as notable contributors. Google Trends also showed positive SHAP contributions, suggesting greater public attention and information-seeking activity in affected regions (Fig. 6c).

As the epidemic entered the region-specific resurgence phase (**Period 4**), reported mpox incidence declined in many regions but persisted in localized hotspots, particularly in the Southern Hemisphere and Africa. Burundi reported the highest incidence rate (27.43 per 100,000) (Fig. 5g-h). SHAP analysis (Fig. 6d) identified GDP per capita, conflict events, and hospital bed availability as major contributors to model-predicted incidence in this phase, indicating that structural vulnerability indicators were strongly associated with reported resurgence patterns.

## Discussion

This study delineated the multi-phase dynamics of global mpox burden and identified four distinct temporal-epidemiological phases, including localized emergence, global spread, sustained dissemination, and regional resurgence. Based on a phase-sensitive analytical framework, we advanced beyond static factor analysis by quantifying how macro-level correlates of mpox burden were reconfigured across epidemic phases. Our findings revealed a pronounced shift from behavioral factors in the early phases to structural vulnerability indicators during the later resurgence, providing a dynamic account of phase-dependent changes in global mpox burden. Furthermore, the LGBT+ context-aware grouping analysis demonstrated that sociocultural and legal environments were associated with regional heterogeneity in reported incidence, highlighting differences in surveillance visibility and reporting completeness.

Previous studies have largely examined either endemic mpox in Africa [60, 61] or the Clade IIb-associated international outbreak in 2022 [62]. Our phase-sensitive analysis provides a comparative perspective by showing that correlates of reported mpox burden differed across epidemic contexts. During the international outbreak, available GISAID genomes were predominantly assigned to Clade IIb, behavior-related variables were more prominent in model interpretation [63], consistent with the concentration of early reported cases in Gay, bisexual, men who have sex with men (GBMSM) contact networks and internationally connected populations [64–67]. The later Africa-centered resurgence presented a different epidemiological context, with the available GISAID genomes showing increased representation of Clades I and subclade Ib. Correspondingly, structural vulnerability indicators emerged as leading model contributors. This pattern aligns with reports that mpox control in Africa is shaped by socioeconomic deprivation, insecurity, limited surveillance, diagnostic constraints, and inequitable access to vaccines and therapeutics [60, 68, 69]. The concurrent rise in reported infections among women, children, and pregnant individuals further suggests a broader case profile than that observed during the early concentration in sexual networks [70, 71]. Together, these findings suggest that the shift from behavioral to structural correlates reflects changing epidemic, lineage, demographic, and health-system contexts underlying reported mpox burden [72].

Moreover, our analysis revealed a context-dependent association between the LGBT+ Legal Index and reported mpox incidence. During the early Clade IIb-associated international outbreak, the positive SHAP contributions observed in high-index settings likely reflected stronger engagement between affected communities and sexual health services, together with more complete case ascertainment. More inclusive legal and sociocultural environments may better support healthcare access and testing uptake, while reduced stigma facilitates disclosure of exposure history and case notification [73, 74]. This interpretation aligns with empirical evidence linking protective policies to higher vaccine uptake [75] and with studies showing stronger risk awareness and intervention adherence among GBMSM communities in supportive environments [76–80]. Conversely, the negative SHAP contributions in more restrictive settings may indicate under-ascertainment arising from surveillance gaps and healthcare barriers, rather than lower underlying burden [81], as stigma, care avoidance, and diagnostic delays reduce diagnosis and reporting [82, 83]. These findings are consistent with previous research demonstrating that structural discrimination and weak health infrastructure can further reduce surveillance sensitivity [84–86]. Consequently, the divergent SHAP directions suggest that the LGBT+ Legal Index captures differences in public health engagement, service access, and reporting completeness across legal and sociocultural contexts.

Based on the phase-stratified findings, our results support several tailored intervention strategies. In earlier phases, when behavioral factors were more prominent, targeted surveillance and risk communication among affected community networks, together with enhanced monitoring in travel-linked settings, may support earlier detection and containment of cross-border spread [87]. Second, as structural vulnerability indicators became more prominent in later phases, global health efforts should strengthen essential health-system capacity in resource-limited settings, including equitable access to diagnostics, vaccines, therapeutics, and routine surveillance [18, 62, 88]. Furthermore, our findings highlight the need for sustained stigma reduction across legal and sociocultural contexts. In legally inclusive settings, sustained trust-based partnerships with LGBT+ communities can help maintain service access and public health engagement [86]. In restrictive environments, confidential testing, non-discriminatory clinical services, and community-led education are critical for reducing barriers to diagnosis and reporting.

Several limitations should be considered. First, case underreporting remains a persistent challenge in infectious disease surveillance, particularly for stigmatized illnesses such as mpox, where social or legal penalties related to same-sex behavior may deepen underestimation and reporting bias [10, 89]. Second, heterogeneity in diagnostic capacity may further affect data completeness, especially in parts of Africa, where deficiencies in testing infrastructure lead to under-ascertainment [87]. Third, reporting delays and aggregation lags may additionally underestimate disease burden and complicate phase-specific interpretation, particularly in resource-limited settings [10]. Furthermore, the WHO surveillance data used as the primary outcome did not consistently assign reported cases to MPXV clades or subclades, preventing reliable clade-stratified modeling. Although available GISAID genomes helped contextualize lineage composition, they were unevenly distributed across regions and phases, and were influenced by sequencing capacity. Therefore, these genomes could not be treated as a representative sampling frame for reported cases or used to estimate clade-specific incidence. In addition, although machine learning models effectively captured complex associations, they could not account for unmeasured confounders such as vaccine-derived immunity owing to data unavailability. The cross-sectional analysis focusing on associations at specific phases of the epidemic did not consider causal relationships or time-series dynamics. Finally, the national-level aggregation may obscure important subnational heterogeneity in both risk factors and transmission dynamics. Future studies would benefit from representative genomic sampling, sentinel surveillance and finer-scale spatial data to support clade-stratified analyses, improve model interpretation, and strengthen causal inference.

## Conclusions

Global reported mpox burden exhibits phase-dependent dynamics, with model-associated correlates shifting from early behavioral factors to later structural vulnerability indicators, particularly socioeconomic disparities and health-system constraints. These epidemiological shifts underscore that mpox control should not rely on a uniform intervention model, but instead requires stage-specific strategies that synchronize behavioral risk mitigation with structural vulnerability reduction. Our analysis provides an evidence base for refining global mpox control strategies and presents a public health framework applicable to similar zoonotic diseases with complex human-animal interface dynamics.

## Supplementary Information

Below is the link to the electronic supplementary material.

Supplementary Material 1: Additional file 1: Tables S1–S13.

Supplementary Material 2: Additional file 2: Figures S1–S12.

Supplementary Material 3: Additional file 3: Texts S1–S3.

## Data Availability

The datasets used for this study are publicly available from the sources cited in the manuscript and listed in Additional file 1: Table S1. All supplementary materials are provided in Additional files 1–3.

## Abbreviations

CatBoost: Categorical Boosting
COVID-19: coronavirus disease 2019
GBDT: Gradient Boosting Decision Tree
GBMSM: gay, bisexual, and other men who have sex with men
GDP: Gross Domestic Product
GISAID: Global Initiative on Sharing All Influenza Data
HIV: Human Immunodeficiency Virus
IHR: International Health Regulations
LGBT+: Lesbian, gay, bisexual, transgender and other sexual and gender minorities
LightGBM: Light Gradient Boosting Machine
MPXV: mpox virus
MSM: Men who have sex with men
PHEIC: Public Health Emergency of International Concern
RFECV: Recursive Feature Elimination with Cross-Validation
SHAP: Shapley Additive Explanations
WHO: World Health Organization
XGBoost: eXtreme Gradient Boosting
