# Supplementary material for: From behavioral to structural correlates: a multi-phase analysis of global reported mpox
Source: BMC Med. 2026 Jul 21;24:484. doi: 10.1186/s12916-026-05068-4 (PMC13563967; doi:10.1186/s12916-026-05068-4)
Supplement: Supplementary file 1 [file 12916_2026_5068_MOESM1_ESM.pdf]

Additional file 1: Tables S1-S13

Table S1. Detailed list of covariates used in the analysis. 1

Table S2. Results of feature selection via recursive feature elimination with cross-validation (RFECV) for the study period..5

Table S3. Results of feature selection via recursive feature elimination with cross-validation (RFECV) for the study period1..6

Table S4. Results of feature selection via recursive feature elimination with cross-validation (RFECV) for the study period2..7

Table S5. Results of feature selection via recursive feature elimination with cross-validation (RFECV) for the study period3..8

Table S6. Results of feature selection via recursive feature elimination with cross-validation (RFECV) for the study period4..9

Table S7. Hyperparameter ranges for different machine learning algorithms. 10

Table S8. Performance metrics and optimized hyperparameters of the best-fitted models for Mpox incidence prediction for different time periods. 11

Table S9. Global and regional surveillance of Mpox cases: A temporal overview of affected countries and confirmed cases (2022.1-2025.4) 13

Table S10. Gender-specific distribution of Mpox cases across WHO regions: Age-group analysis (2022.1-2025.4)...14

Table S11. Distribution of reported mpox cases and available GISAID MPXV genomes by epidemic phase (2022.1-2025.4)...15

Table S12. Mpox exposure pathways and clinical outcomes: A statistical analysis of risk factors and case distribution (2022.1-2025.4)..16

Table S13. Comparative evaluation of ensemble machine learning models for Mpox transmission prediction: training and testing Metrics (R^2, MAE, RMSE) across temporal outbreak phases..17

Table S1. Detailed list of covariates used in the analysis.

Domain	Variables	Type	Temporal	Definition	Data source	Missing	Median [Q1–Q3]
Socio-demographic	Population Size	Structural	2023	Population by country	UN Population Division [26]	0/133	10644855 (3185071-37712517)
	Population Density	Structural	2023	The number of people per km² of land area	UN Population Division [26]	0/133	105.20 (42.77-250.21)
	Age under 15	Structural	2023	Population, ages 0-14	UN Population Division [26]	0/133	0.20 (0.16-0.29)
	Age 15-24	Structural	2023	Population, ages 15-24	UN Population Division [26]	0/133	0.13 (0.11-0.18)
	Age 25-64	Structural	2023	Population, ages 25-64	UN Population Division [26]	0/133	0.52 (0.46-0.55)
	Age 65 +	Structural	2023	Population, ages 65 and above	UN Population Division [26]	0/133	0.12 (0.05-0.19)
	Sex Ratio	Structural	2023	The number of male population per 100 female population	UN Population Division [26]	0/133	97.92 (94.60-100.45)
	MSM Population	Behavioral	2019	Adolescents and young men who have sex with men: Population size estimate	UNAIDS [27]	14/133	26900 (10600-57200)
	Sex Worker	Behavioral	2019	Adolescents and young sex workers: Population size estimate	UNAIDS [27]	14/133	13320 (2700-29200)
Socioeconomic	GDP	Structural	2022	GDP per capita	The World Bank [28]	15/133	18827.78 (8370.73-40223.01)
	Poverty Rate	Structural	2023	Share of population living in extreme poverty.	The World Bank [28]	22/133	0.23 (0.14-0.36)
	Urban population	Structural	2022	Share of the population living in urban areas	The World Bank [28]	4/133	68.74 (52.66-84.73)

Domain	Variables	Type	Temporal	Definition	Data source	Missing	Median [Q1–Q3]
Socio-cultural	LGBT+ Legal	Structural	2024	The LGBT legal index measures the legal status of 13 different issues	Equaldex [29]	10/133	55 (33.6-81)
	LGBT+ Public Opinion	Structural	2024	The LGBT public opinion index measures the public attitudes towards LGBT people using surveys and polls from reputable organizations	Equaldex [29]	13/133	36 (21-56)
	GDI	Structural	2022	Gender Development Index	United Nations Development Programme [30]	15/133	0.97 (0.93-0.99)
	Tertiary Education Rate	Structural	2020	Percentage of the population aged 25 to 65 years who have either completed or partially completed tertiary education	Our World in Data [31]	25/133	14.7 (6.1-26.5)
	Average Schooling	Structural	2020	Average years of formal education for individuals aged 15-64	Our World in Data [32]	25/133	9.12 (6.87-11.57)
	Google Trend	Behavioral	2022-2024	The search popularity index of Mpox in various countries around the world between January 2022 and October 2024	Google Trend	0/133	7.82 (3.6-23.02)
Healthcare	Total Health Expenditure	Structural	2021	Total healthcare expenditure as the share of national gross domestic product (GDP)	World Health Organization [33]	11/133	7.41 (4.81-9.38)
	Gov Health Expenditure	Structural	2021	Domestic general government health expenditure expressed as a percentage of total expenditure of the general government	World Health Organization [34]	12/133	3.75 (2.03-5.89)
	UHCI	Structural	2021	The Universal Health Service Coverage Index	World Health Organization [35]	11/133	74.55 (56.79-81.77)

Domain	Variables	Type	Temporal	Definition	Data source	Missing	Median [Q1–Q3]
	IHR	Structural	2021	Average of 15 International Health Regulations core capacity scores, PAR version, 2nd edition	World Health Organization [36]	12/133	66 (54-81)
	Hospital Beds	Structural	2019	Hospital beds per 1,000 people	The World Bank [28]	10/133	2.52 (1.38-4.49)
	Physicians	Structural	2021	Medical doctors per 1,000 people	The World Bank [28]	6/133	2.36 (0.79-3.65)
**Disease Burden**	HIV Prevalence	Behavioral	2021	HIV Prevalence	2021 Global Burden of Disease Study [37]	8/133	0.002 (0.0006-0.01)
	STI	Behavioral	2021	Sexually Transmitted Infections	2021 Global Burden of Disease Study [37]	8/133	0.19 (0.13-0.25)
	Alcohol Use Disorders	Structural	2021	Alcohol Use Disorders	2021 Global Burden of Disease Study [37]	8/133	0.0014 (0.0007-0.0028)
	Drug Use Disorders	Structural	2021	Drug Use Disorders	2021 Global Burden of Disease Study [37]	8/133	0.0004 (0.0002-0.0011)
	Maternal Disorders	Structural	2021	Maternal Disorders	2021 Global Burden of Disease Study [37]	8/133	0.0009 (0.0006-0.0016)
	Neonatal Disorders	Structural	2021	Neonatal Disorders	2021 Global Burden of Disease Study [37]	8/133	0.017 (0.013-0.019)
	COVID-19	Structural	2022-2024	Cumulative COVID-19 cases	Our World in Data [38]	1/133	305093 (45161.4-2308236)
	Conflicts	Structural	2023	Death rate in armed conflicts based on where they occurred	Our World in Data [39]	9/133	0 (0-0.031)
	Nutritional Deficiencies	Structural	2021	Nutritional Deficiencies	2021 Global Burden of Disease Study [37]	8/133	0.16 (0.09-0.25)

Domain	Variables	Type	Temporal	Definition	Data source	Missing	Median [Q1–Q3]
**Transportation**	Air Passengers	Behavioral	2021	Domestic and international passengers of airlines registered in the country.	The World Bank [28]	29/133	1276465 (339648.2-8729635)
	Inbound Arrivals	Behavioral	2022	Trips by people who arrive from abroad and stay overnight	The United Nations World Tourism Organization [40]	10/133	1937000 (614800-5698000)
	Road Density	Structural	2018	Global road density raster, total density, all types combined	GLOBIO [41]	3/133	275.60 (126.5-677.31)
**Environmental Sanitation**	Average Temperature	Structural	2023	Average monthly surface temperature	ERA5 [42]	0/133	22.06 (11.77-26.38)
	Annual precipitation	Structural	2023	Total annual precipitation	ERA5 [42]	0/133	1072.60 (679.23-1607.40)
	PM2.5	Structural	2019	Exposure to particulate matter air pollution (PM2.5)	ERA5 [42]	0/133	10.59 (8.24-14.54)
	Treated Wastewater	Structural	2022	Share of domestic wastewater that is safely treated	United Nations Human Settlements Programme [43]	38/133	43.41 (24.61-82.05)
	Sanitation Coverage	Structural	2022	Share of the population using safely managed sanitation facilities	World Health Organization /UNICEF [44]	39/133	52.14 (31.97-87.84)

Table S2. Results of feature selection via recursive feature elimination with cross-validation (RFECV) for the study period.

Model	Mean RMSE	Number	Features
XGBoost	4.6668	32	Population Size, Population Density, Age under 15, Age 15-24, Age 25-64, Sex Ratio, MSM Population, GDP, Urban Population, LGBT+ Legal, LGBT+ Public Opinion, GDI, Tertiary Education Rate, Google Trend, Total Health Expenditure, Gov Health Expenditure, UHCI, IHR, HIV Prevalence, STI, Alcohol Use Disorders, Maternal Disorders, Neonatal Disorders, COVID, Nutritional Deficiencies, Air Passengers, Inbound Arrivals, Average Temperature, Annual precipitation, PM2.5, Treated Wastewater, Sanitation Coverage
RF	4.0678	15	Population Size, Population Density, Age under 15, Age 25-64, GDP, Poverty Rate, Urban Population, LGBT+ Legal, Total Health Expenditure, IHR, HIV Prevalence, COVID, Nutritional Deficiencies, Inbound Arrivals, Sanitation Coverage
LightGBM	3.9415	25	Population Density, Age 65 +, Sex Ratio, MSM Population, Sex Worker, Poverty Rate, Urban Population, LGBT+ Legal, LGBT+ Public Opinion, Average Schooling, Google Trend, Total Health Expenditure, Gov Health Expenditure, IHR, Hospital Beds, Physicians, HIV Prevalence, STI, COVID, Conflicts, Nutritional Deficiencies, Air Passengers, Road Density, Average Temperature, Sanitation Coverage
GBDT	4.2191	22	Population Size, Population Density, Age under 15, Age 25-64, Sex Ratio, Sex Worker, GDP, Poverty Rate, Urban Population, LGBT+ Legal, LGBT+ Public Opinion, GDI, Total Health Expenditure, UHCI, IHR, HIV Prevalence, STI, COVID, Nutritional Deficiencies, Air Passengers, Inbound Arrivals, Sanitation Coverage
**CatBoost**	**3.6982**	**22**	**Population Size, Population Density, Age under 15, Age 25-64, Sex Ratio, Sex Worker, GDP, Urban Population, LGBT+ Legal, Average Schooling, Google Trend, Total Health Expenditure, UHCI, IHR, HIV Prevalence, Alcohol Use Disorders, Neonatal Disorders, Nutritional Deficiencies, Air Passengers, Inbound Arrivals, Annual precipitation, Sanitation Coverage**

Table S3. Results of feature selection via recursive feature elimination with cross-validation (RFECV) for the study period1.

Model	Mean RMSE	Number	Features
XGBoost	0.198	19	Population Size, Population Density, Age under 15, Age 15-24, Age 25-64, Sex Ratio, GDP, Poverty Rate, Urban Population, LGBT+ Legal, Google Trend, Gov Health Expenditure, UHCI, Hospital Beds, Physicians, Alcohol Use Disorders, Neonatal Disorders, Road Density, Average Temperature
RF	0.1171	22	Population Size, Population Density, Age under 15, Age 65 +, Sex Ratio, MSM Population, Poverty Rate, LGBT+ Public Opinion, GDI, Tertiary Education Rate, Google Trend, UHCI, Physicians, STI, Maternal Disorders, Neonatal Disorders, Conflicts, Inbound Arrivals, Road Density, Average Temperature, Annual precipitation, Treated Wastewater
LightGBM	0.1277	15	Alcohol Use Disorders, Drug Use Disorders, Maternal Disorders, Neonatal Disorders, COVID, Conflicts, Nutritional Deficiencies, Air Passengers, Inbound Arrivals, Road Density, Average Temperature, Annual precipitation, PM2.5, Treated Wastewater, Sanitation Coverage
**GBDT**	**0.111**	**26**	**Population Size, Population Density, Age 15-24, Age 25-64, Sex Ratio, Sex Worker, Poverty Rate, LGBT+ Legal, LGBT+ Public Opinion, Tertiary Education Rate, Google Trend, UHCI, IHR, STI, Alcohol Use Disorders, Drug Use Disorders, Neonatal Disorders, COVID, Nutritional Deficiencies, Air Passengers, Inbound Arrivals, Road Density, Average Temperature, Annual precipitation, Treated Wastewater, Sanitation Coverage**
CatBoost	0.1181	34	Population Size, Population Density, Age under 15, Age 15-24, Age 65 +, Sex Ratio, MSM Population, Sex Worker, GDP, Poverty Rate, Urban Population, LGBT+ Legal, LGBT+ Public Opinion, GDI, Tertiary Education Rate, Average Schooling, Google Trend, Total Health Expenditure, UHCI, IHR, Physicians, HIV Prevalence, Alcohol Use Disorders, Drug Use Disorders, Maternal Disorders, Neonatal Disorders, COVID, Conflicts, Inbound Arrivals, Road Density, Average Temperature, Annual precipitation, PM2.5, Treated Wastewater

Table S4. Results of feature selection via recursive feature elimination with cross-validation (RFECV) for the study period2.

Model	Mean RMSE	Number	Features
XGBoost	2.9814	23	Population Size, Population Density, Age under 15, Age 25-64, Sex Ratio, MSM Population, Urban Population, LGBT+ Legal, Tertiary Education Rate, Total Health Expenditure, Gov Health Expenditure, UHCI, IHR, Hospital Beds, HIV Prevalence, Alcohol Use Disorders, Nutritional Deficiencies, Air Passengers, Inbound Arrivals, Average Temperature, Annual precipitation, Treated Wastewater, Sanitation Coverage
RF	2.6676	35	Population Size, Population Density, Age under 15, Age 25-64, Age 65 +, Sex Ratio, MSM Population, Sex Worker, GDP, Urban Population, LGBT+ Legal, LGBT+ Public Opinion, GDI, Tertiary Education Rate, Google Trend, Total Health Expenditure, Gov Health Expenditure, UHCI, IHR, Physicians, HIV Prevalence, STI, Alcohol Use Disorders, Maternal Disorders, Neonatal Disorders, COVID, Nutritional Deficiencies, Air Passengers, Inbound Arrivals, Road Density, Average Temperature, Annual precipitation, PM2.5, Treated Wastewater, Sanitation Coverage
LightGBM	2.8306	28	Population Size, Population Density, Age 65 +, Sex Ratio, GDP, Poverty Rate, Urban Population, LGBT+ Legal, LGBT+ Public Opinion, Google Trend, Total Health Expenditure, Gov Health Expenditure, UHCI, Hospital Beds, Physicians, HIV Prevalence, STI, Alcohol Use Disorders, Drug Use Disorders, COVID, Nutritional Deficiencies, Air Passengers, Road Density, Average Temperature, Annual precipitation, PM2.5, Treated Wastewater, Sanitation Coverage
GBDT	2.6124	38	Population Size, Population Density, Age 15-24, Age 25-64, Sex Ratio, MSM Population, Sex Worker, GDP, Poverty Rate, Urban Population, LGBT+ Legal, LGBT+ Public Opinion, GDI, Tertiary Education Rate, Average Schooling, Google Trend, Total Health Expenditure, Gov Health Expenditure, UHCI, IHR, Hospital Beds, Physicians, HIV Prevalence, STI, Alcohol Use Disorders, Drug Use Disorders, Maternal Disorders, Neonatal Disorders, COVID, Nutritional Deficiencies, Air Passengers, Inbound Arrivals, Road Density, Average Temperature, Annual precipitation, PM2.5, Treated Wastewater, Sanitation Coverage
**CatBoost**	**2.5261**	**19**	**Population Size, Population Density, Sex Ratio, GDP, Urban Population, LGBT+ Legal, Total Health Expenditure, UHCI, HIV Prevalence, STI, Alcohol Use Disorders, Drug Use Disorders, COVID, Nutritional Deficiencies, Air Passengers, Inbound Arrivals, Annual precipitation, PM2.5, Sanitation Coverage**

Table S5. Results of feature selection via recursive feature elimination with cross-validation (RFECV) for the study period3.

Model	Mean RMSE	Number	Features
XGB	0.3464	29	Population Size, Population Density, Age under 15, Age 15-24, Age 25-64, Age 65 +, Sex Ratio, Sex Worker, GDP, Poverty Rate, LGBT+ Legal, LGBT+ Public Opinion, GDI, Tertiary Education Rate, Average Schooling, UHCI, IHR, Hospital Beds, HIV Prevalence, STI, Alcohol Use Disorders, Maternal Disorders, Conflicts, Air Passengers, Inbound Arrivals, Road Density, Average Temperature, Annual precipitation, PM2.5
RF	0.3734	23	Population Density, Age under 15, Age 65 +, Sex Ratio, Sex Worker, GDP, Poverty Rate, LGBT+ Public Opinion, GDI, Tertiary Education Rate, Google Trend, UHCI, Physicians, HIV Prevalence, STI, Alcohol Use Disorders, Maternal Disorders, Nutritional Deficiencies, Inbound Arrivals, Road Density, Annual precipitation, Treated Wastewater, Sanitation Coverage
LightGBM	0.3808	25	Population Density, Age 65 +, Sex Ratio, GDP, LGBT+ Legal, LGBT+ Public Opinion, GDI, Tertiary Education Rate, Population Density, Age 65 +, Sex Ratio, GDP, LGBT+ Public Opinion, GDI, Tertiary Education Rate, Average Schooling, Google Trend, Total Health Expenditure, UHCI, HIV Prevalence, STI, Alcohol Use Disorders, Maternal Disorders, Neonatal Disorders, COVID, Conflicts, Air Passengers, Road Density, Average Temperature, Annual precipitation, PM2.5, Treated Wastewater, Sanitation Coverage
GBDT	0.3441	17	Sex Ratio, MSM Population, GDP, Poverty Rate, GDI, Google Trend, UHCI, IHR, HIV Prevalence, Alcohol Use Disorders, Neonatal Disorders, Nutritional Deficiencies, Air Passengers, Inbound Arrivals, Road Density, Treated Wastewater, Sanitation Coverage
**CatBoost**	**0.3049**	**32**	**Population Size, Age under 15, Age 25-64, Age 65 +, Sex Ratio, MSM Population, Sex Worker, GDP, Poverty Rate, LGBT+ Legal, LGBT+ Public Opinion, GDI, Tertiary Education Rate, Average Schooling, Google Trend, Total Health Expenditure, UHCI, IHR, Hospital Beds, Physicians, HIV Prevalence, STI, Alcohol Use Disorders, Maternal Disorders, Neonatal Disorders, COVID, Conflicts, Air Passengers, Inbound Arrivals, Road Density, Annual precipitation, Sanitation Coverage**

Table S6. Results of feature selection via recursive feature elimination with cross-validation (RFECV) for the study period4.

Model	Mean RMSE	Number	Features
XGB	3.2092	15	Population Size, Population Density, Age under 15, Age 25-64, GDP, Poverty Rate, Urban Population, LGBT+ Legal, GDI, Tertiary Education Rate, Average Schooling, Google Trend, Total Health Expenditure, Physicians, Nutritional Deficiencies
RF	2.3819	38	Population Size, Population Density, Age under 15, Age 15-24, Age 25-64, Age 65 +, Sex Ratio, MSM Population, GDP, Poverty Rate, Urban Population, LGBT+ Legal, GDI, Average Schooling, Google Trend, Total Health Expenditure, Gov Health Expenditure, UHCI, IHR, Hospital Beds, Physicians, HIV Prevalence, STI, Alcohol Use Disorders, Drug Use Disorders, Maternal Disorders, Neonatal Disorders, COVID, Conflicts, Nutritional Deficiencies, Air Passengers, Inbound Arrivals, Road Density, Average Temperature, Annual precipitation, PM2.5, Treated Wastewater, Sanitation Coverage
LightGBM	2.9625	33	Age under 15, Age 15-24, Age 25-64, Age 65 +, Sex Ratio, MSM Population, Poverty Rate, LGBT+ Legal, LGBT+ Public Opinion, GDI, Tertiary Education Rate, Average Schooling, Google Trend, Total Health Expenditure, Gov Health Expenditure, UHCI, IHR, Hospital Beds, Physicians, HIV Prevalence, STI, Alcohol Use Disorders, Neonatal Disorders, COVID, Conflicts, Nutritional Deficiencies, Air Passengers, Inbound Arrivals, Road Density, Average Temperature, Annual precipitation, Treated Wastewater, Sanitation Coverage
GBDT	2.7143	18	Population Density, Age 25-64, Sex Ratio, Sex Worker, GDP, LGBT+ Legal, GDI, Average Schooling, Google Trend, Total Health Expenditure, UHCI, Physicians, Nutritional Deficiencies, Inbound Arrivals, Road Density, Average Temperature, Annual precipitation, Treated Wastewater
**CatBoost**	**2.2928**	**21**	**Population Size, Population Density, Age under 15, Age 25-64, Sex Ratio, Sex Worker, GDP, Urban Population, LGBT+ Legal, Average Schooling, Google Trend, Hospital Beds, Alcohol Use Disorders, Drug Use Disorders, Neonatal Disorders, Conflicts, Nutritional Deficiencies, Inbound Arrivals, Average Temperature, Treated Wastewater, Sanitation Coverage**

Table S7. Hyperparameter ranges for different machine learning algorithms.

Algorithm	Hyperparameters
**XGBoost**	learning_rate: (0.0001, 1)<br>max_depth:(3, 12)<br>min_child_weight:(1, 10)<br>gamma:(0,5)<br>subsample: (0.5, 1.0)<br>colsample_bytree: (0.5, 1.0)<br>n_estimators:(100, 1000)
**RandomForest**	max_depth: (3, 12)<br>min_samples_split: (2, 20)<br>min_samples_leaf:(1,10)<br>n_estimators: (100, 1000)
**LightGBM**	num_leaves: (2, 100)<br>learning_rate: (0.0001, 1)<br>max_depth: (3, 12)<br>min_data_in_leaf: (1, 20)<br>feature_fraction: 0.5, 1.0)<br>bagging_fraction: (0.5, 1.0)<br>bagging_freq: (5, 10)<br>lambda_l1: (0.0, 10.0)<br>lambda_l2: (0.0, 10.0)<br>min_gain_to_split:(0.001, 0.1)<br>n_estimators: (100, 1000)
**GBDT**	max_depth: (3, 12)<br>learning_rate: (0.0001, 1)<br>min_samples_split: (2, 20)<br>min_samples_leaf: (1, 10)<br>subsample:(0.5, 1.0)<br>n_estimators: (100, 1000)
**CatBoost**	depth: (3, 12)<br>learning_rate:(0.0001, 1)<br>l2_leaf_reg:(1, 10)<br>random_strength:(0.1,1)<br>iterations: (100, 1000)

Table S8. Performance metrics and optimized hyperparameters of the best-fitted models for Mpox incidence prediction for different time periods.

	Model Name	Best Params	Train R2	Train MAE	Train RMSE	Test R2	Test MAE	Test RMSE
**Period**	**LightGBM**	num leaves: 20<br>n estimators:590<br>learning rate: 0.450<br>max depth: 4<br>min data in leaf: 2<br>feature fraction: 0.797<br>bagging fraction: 0.850<br>bagging freq: 10<br>lambda l1: 0.805<br>lambda l2: 0.518<br>min gain to split: 0.002	0.998	0.107	0.169	0.843	1.373	1.682
**Period1**	**CatBoost**	iterations: 975<br>learning rate: 0.942<br>depth: 4<br>l2 leaf reg: 6.733<br>random strength: 0.929	1.000	2.29E-09	4.70E-09	0.665	0.015	0.021
**Period2**	**GBDT**	n estimators: 104<br>max depth: 10<br>learning rate: 0.623<br>min samples split: 17	1.000	1.04E-08	1.38E-08	0.681	1.372	1.994

	Model Name	Best Params	Train R2	Train MAE	Train RMSE	Test R2	Test MAE	Test RMSE
		min samples leaf: 2<br>subsample:0.843						
**Period3**	**CatBoost**	iterations: 1000<br>learning rate: 0.764<br>depth: 10<br>l2 leaf reg: 5.952<br>random strength: 0.653	1.000	8.00E-09	2.19E-08	0.766	0.106	0.141
**Period4**	**LightGBM**	num leaves: 33<br>n estimators: 887<br>learning rate: 0.146<br>max depth: 5<br>min data in leaf: 4<br>feature fraction: 0.953<br>bagging fraction: 0.984<br>bagging freq: 6<br>lambda l1: 0.594<br>lambda l2: 8.783<br>min gain to split: 0.003	0.998	0.080	0.177	0.799	0.923	1.189

Table S9. Global and regional surveillance of Mpox cases: A temporal overview of affected countries and confirmed cases (2022.1-2025.4)

	Period1 2022/1/1-2022/5/28		Period2 2022/5/29-2023/5/11		Period3 2023/5/12-2024/8/13		Period4 2024/8/14-2025/4/30		Period 2022/1/1-2025/4/30			
	**Affected Countries**	**Confirmed Cases**	**Affected Countries**	**Confirmed Cases**	**Affected Countries**	**Confirmed Cases**	**Affected Countries**	**Confirmed Cases**	**Affected Countries**	**Confirmed Cases**	**Detailed Confirmed Cases**	**Reporting completeness**
**World**	30	522	110	87565	80	17414	88	33329	138830	133	101709	73.26%
**African region**	6	58	10	1599	13	5188	23	25667	32512	25	672	2.07%
**Region of the Americas**	5	152	30	59740	18	5557	15	3391	68840	31	66935	97.23%
**Eastern Mediterranean Region**	1	4	11	95	6	769	8	39	907	12	126	13.89%
**European region**	17	306	45	25581	27	1793	30	2218	29898	47	29704	99.35%
**South-East Asia Region**	0	0	4	50	5	890	3	88	1028	5	1020	99.22%
**Western pacific region**	1	2	10	500	11	3217	9	1926	5645	13	3252	57.61%

Table S10. Gender-specific distribution of Mpox cases across WHO regions: Age-group analysis (2022.1-2025.4)

Region	Gender	0-9	10-19	20-29	30-39	40-49	50-59	60-69	70-79	80+	All
AFRO	Male	1	11	495	817	471	191	60	8	0	2054
	Female	0	0	17	8	2	1	0	0	0	28
AMRO	Male	220	961	17105	24393	10760	3781	674	77	9	57980
	Female	161	242	881	699	421	194	51	23	11	2683
EMRO	Male	0	4	35	46	18	4	1	0	0	108
	Female	3	1	6	3	1	0	0	0	0	14
EURO	Male	19	205	5990	11676	7539	2848	699	79	10	29065
	Female	14	32	154	159	107	24	7	6	0	503
SEARO	Male	2	4	276	375	183	32	6	1	0	879
	Female	1	0	15	13	7	0	2	0	0	38
WPRO	Male	1	40	917	1236	425	81	15	1	0	2716
	Female	0	2	11	4	5	0	1	1	0	24
World	Male	243	1225	24818	38543	19396	6937	1455	166	19	92802
	Female	179	277	1084	886	543	219	61	30	11	3290
	All	422	1502	25902	39429	19939	7156	1516	196	30	96092

Table S11. Distribution of reported mpox cases and available GISAID MPXV genomes by epidemic phase (2022.1-2025.4)

Phase	Reported cases	Available GISAID genomes	Countries with reported cases	Countries with available genomes	Ia (%)	Ib (%)	IIa (%)	IIb (%)
Period 1	522	298	30	26	11 (3.7)	0 (0.0)	0 (0.0)	287 (96.3)
Period 2	87,565	9,208	110	60	219 (2.4)	0 (0.0)	4 (<0.1)	8,985 (97.6)
Period 3	17,414	2,625	80	49	403 (15.4)	254 (9.7)	6 (0.2)	1,962 (74.7)
Period 4	33,329	3,699	88	42	1,168 (31.6)	1,338 (36.2)	25 (0.7)	1,168 (31.6)

Note: Percentages for subclades were calculated using the number of available GISAID genomes within each phase as the denominator. These data describe available genome composition and should not be interpreted as sequencing coverage or clade-specific incidence.

Table S12. Mpox exposure pathways and clinical outcomes: A statistical analysis of risk factors and case distribution (2022.1-2025.4)

	Yes	No	Unknown or Missing Value	Yes Percent	No Percent
**Men who have sex with men**	32119	4944	97583	0.867	0.133
**Persons living with HIV**	19492	18374	96780	0.515	0.485
**Health worker**	1516	39353	93777	0.037	0.963
**Travel History**	5110	25209	104327	0.169	0.831
**Sexual Transmission**	20760	2727	111159	0.884	0.116
**Hospitalized**	6713	58493	69440	0.103	0.897
**ICU**	50	11644	122952	0.004	0.996
**Died**	204	61357	73085	0.003	0.997

Table S13. Comparative evaluation of ensemble machine learning models for Mpox transmission prediction: training and testing Metrics (R^2, MAE, RMSE) across temporal outbreak phases.

Time	Model Name	Train R2	Train MAE	Train RMSE	Test R2	Test MAE	Test RMSE
	XGBoost	0.989	0.346	0.457	0.674	1.847	2.422
	RandomForest	0.708	1.189	2.341	0.412	1.776	3.252
**Period**	**LightGBM**	**0.998**	**0.107**	**0.169**	**0.843**	**1.373**	**1.682**
	GBDT	1.000	0.000	0.000	0.659	1.701	2.475
	CatBoost	1.000	0.000	0.000	0.746	1.422	2.138
	XGBoost	-0.096	0.081	0.165	0.000	0.033	0.036
	RandomForest	0.505	0.072	0.111	-1.163	0.048	0.054
**Period1**	LightGBM	0.000	0.104	0.158	-1.805	0.051	0.061
	GBDT	1.000	0.000	0.000	0.664	0.016	0.021
	**CatBoost**	**1.000**	**0.000**	**0.000**	**0.665**	**0.015**	**0.021**
	XGBoost	0.960	0.412	0.595	0.551	1.094	2.364
	RandomForest	0.903	0.577	0.929	0.222	1.660	3.113
**Period2**	LightGBM	0.998	0.082	0.144	0.447	1.283	2.624
	**GBDT**	**1.000**	**0.000**	**0.000**	**0.681**	**1.372**	**1.994**
	CatBoost	1.000	0.000	0.000	0.366	1.321	2.809
	XGBoost	0.327	0.310	0.574	0.234	0.208	0.255
	RandomForest	0.243	0.301	0.608	-0.205	0.279	0.320
**Period3**	LightGBM	0.110	0.333	0.659	0.120	0.241	0.274
	GBDT	1.000	0.000	0.000	-0.425	0.245	0.348
	**CatBoost**	**1.000**	**0.000**	**0.000**	**0.766**	**0.106**	**0.141**
	XGBoost	0.920	0.651	1.073	0.762	0.965	1.294
	RandomForest	0.229	1.300	3.327	0.216	1.411	2.346
**Period4**	**LightGBM**	**0.998**	**0.080**	**0.177**	**0.799**	**0.923**	**1.189**
	GBDT	0.930	0.345	1.002	0.739	1.019	1.355
	CatBoost	1.000	0.000	0.000	0.740	0.813	1.352
