# Supplementary material for: From behavioral to structural correlates: a multi-phase analysis of global reported mpox
Source: BMC Med. 2026 Jul 21;24:484. doi: 10.1186/s12916-026-05068-4 (PMC13563967; doi:10.1186/s12916-026-05068-4)

## Additional file 2: Figs S1-S12

Fig S1. COVID-19 cases. (a)Period (b) Period1 (c) Period2 (d) Period3 (e) Period4...1

Fig S2. Google Trend. (a)Period (b) Period1 (c) Period2 (d) Period3 (e) Period4........2

Fig S3. Scatter plot comparison of original and imputed values for variables with missing data (Part 1). ..........................................................................................3

Fig S4. Scatter plot comparison of original and imputed values for variables with missing data (Part 2). ..........................................................................................4

Fig S5. Kernel density comparison of original and imputed values for variables with missing data (Part1). ..........................................................................................5

Fig S6. Kernel density comparison of original and imputed values for variables with missing data (Part2). ..........................................................................................6

Fig S7. Cross-validation RMSE for Recursive Feature Elimination with Cross-Validation (RFECV) across different feature selections (a) Period (b)Period 1 (c)Period 2 (d)Period 3 (e) Period 4..........................................................................................7

Fig S8. Multicollinearity filtering based on correlation and feature importance. (a) Period (c)Period1 (d) Period2 Correlation matrix of features selected through RFECV. (b) Period (d)Period1 (e) Period2 Feature importance ranking obtained from regressor model. Features highlighted in red were excluded due to multicollinearity. .................8

Fig S9. Multicollinearity filtering based on correlation and feature importance. (a) Period3 (c)Period4 Correlation matrix of features selected through RFECV. (b) Period3 (d)Period4 Feature importance ranking obtained from regressor model. Features highlighted in red were excluded due to multicollinearity............................................9

Fig S10. Spatiotemporal distribution and lineage composition of genotyped mpox counts across epidemic phases. (a) Geographic distribution of MPXV clades (Ia, Ib, IIa, and IIb). Pie charts represent the cumulative proportion of clades in each country, with the radius proportional to the total number of genotyped counts. (b) MPXV clade composition across four epidemic periods (Periods 1-4). Reporting countries are grouped by World Health Organization (WHO) regions. Each pie chart illustrates the lineage distribution for a specific country during a given period, with circle sizes reflecting the corresponding number of genotyped counts.........................................10

Fig S11. Regional top five factors of Mpox incidence based on SHAP importance (grouped by WHO regions). ..........................................................................................11

Fig S12. Scatter plots comparing predicted and observed Mpox incidence rates for training (red) and testing (blue) datasets across four outbreak phases. (a) Period 1, (b) Period 2, (c)Period 3, and (d) Period 4. ......................................................................12

**Fig S1. COVID-19 cases. (a)Period (b) Period1 (c) Period2 (d) Period3 (e) Period4.**

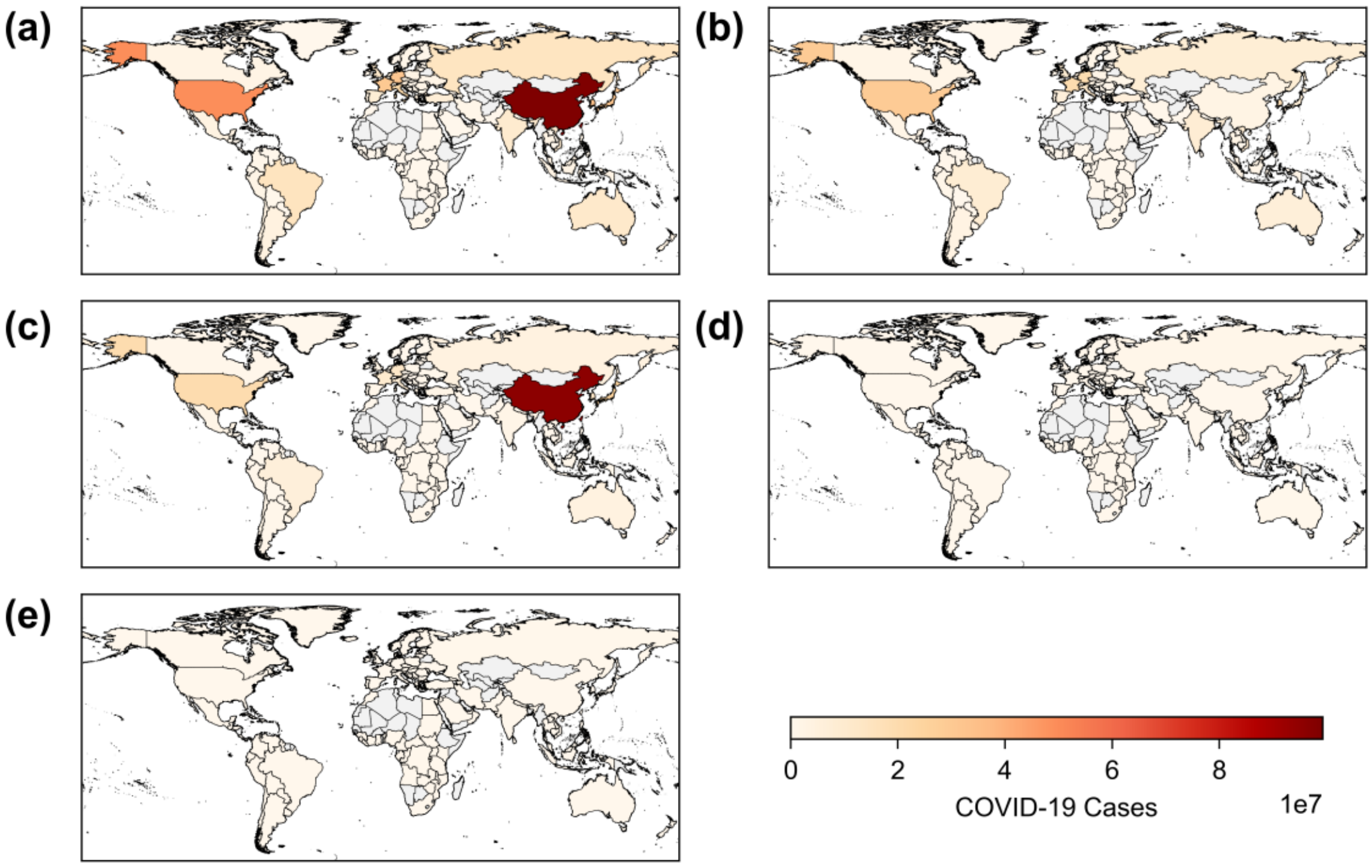

**Fig S2. Google Trend. (a)Period (b) Period1 (c) Period2 (d) Period3 (e) Period4**

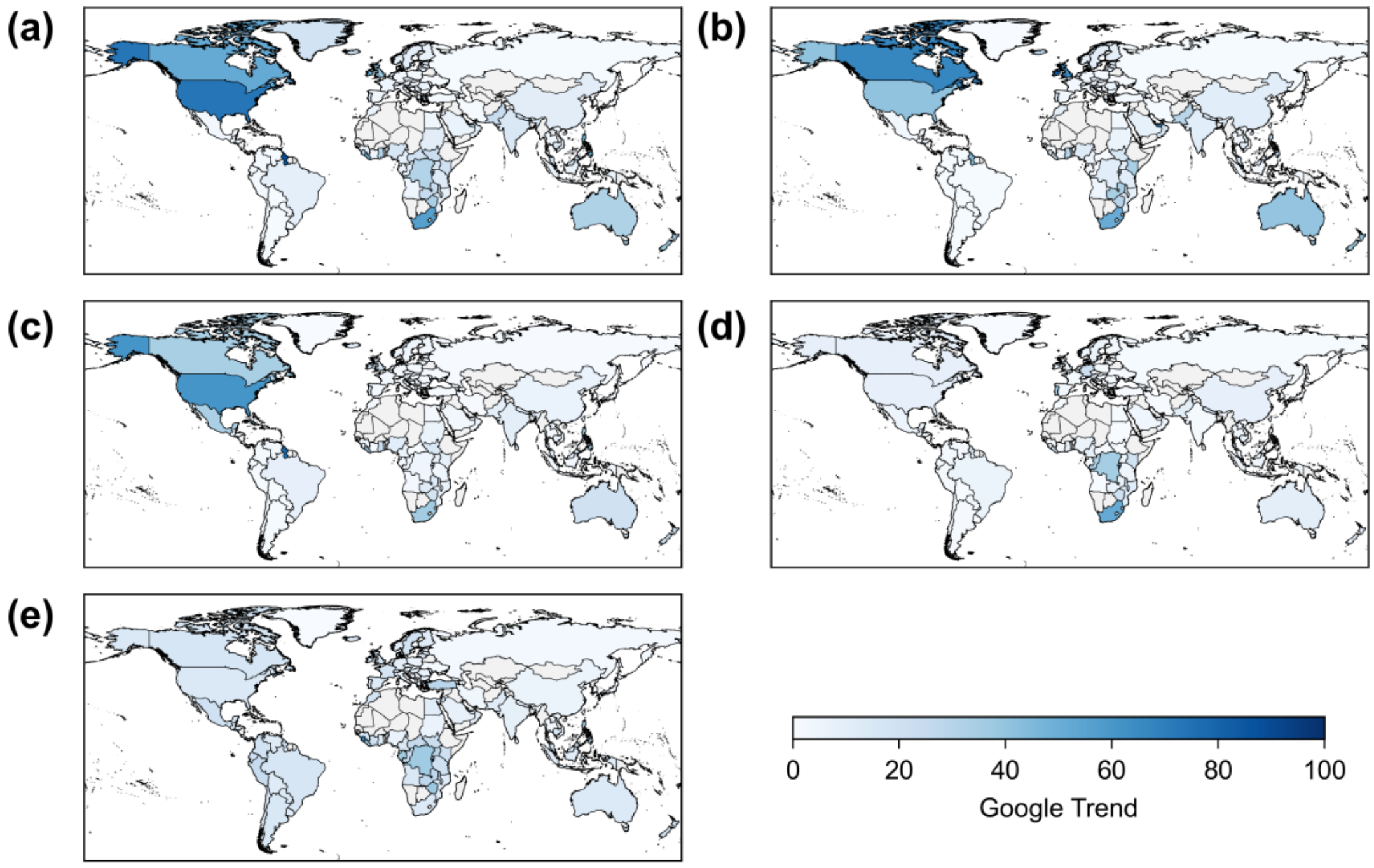

**Fig S3. Scatter plot comparison of original and imputed values for variables with missing data (Part 1).**

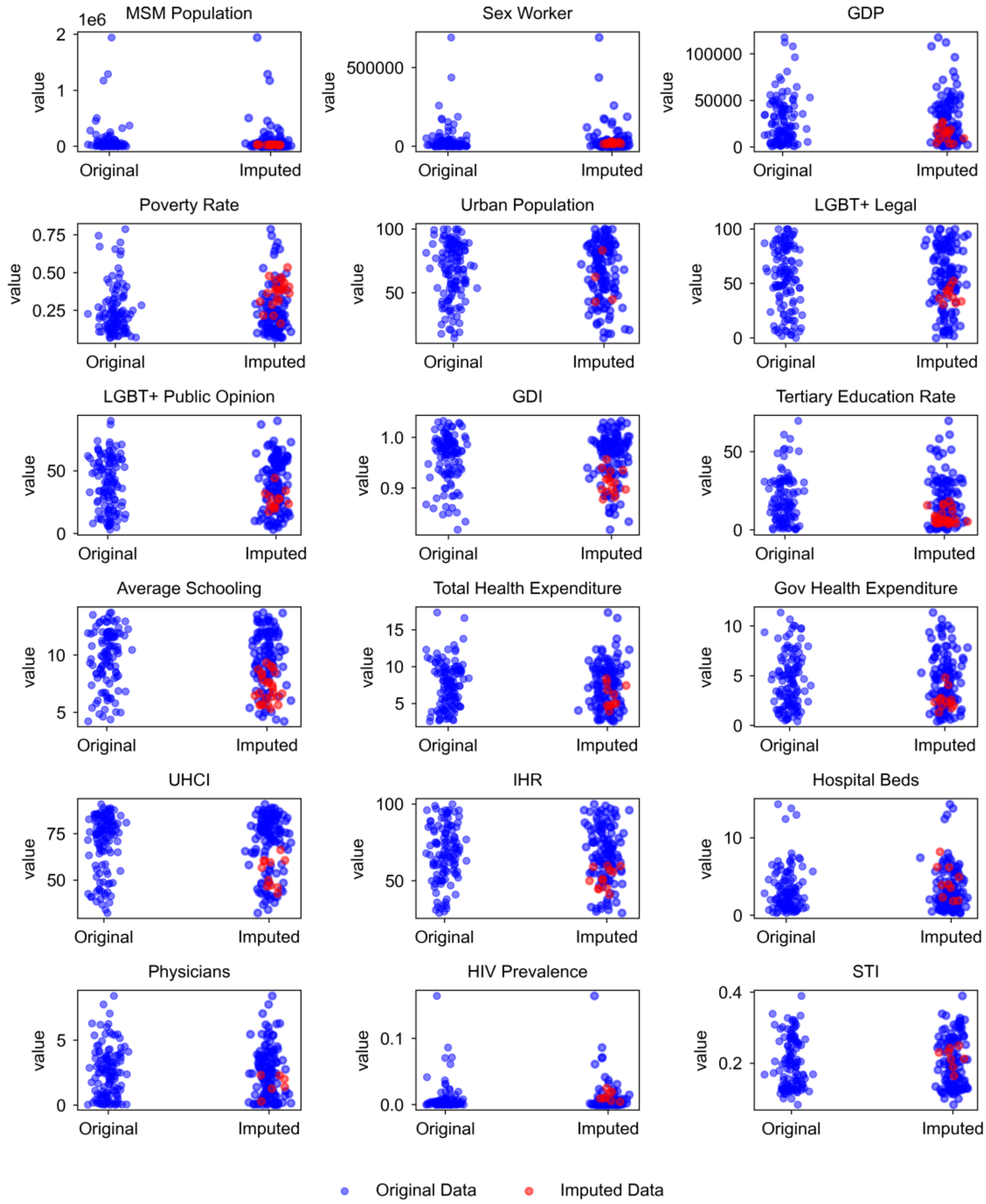

**Fig S4. Scatter plot comparison of original and imputed values for variables with missing data (Part 2).**

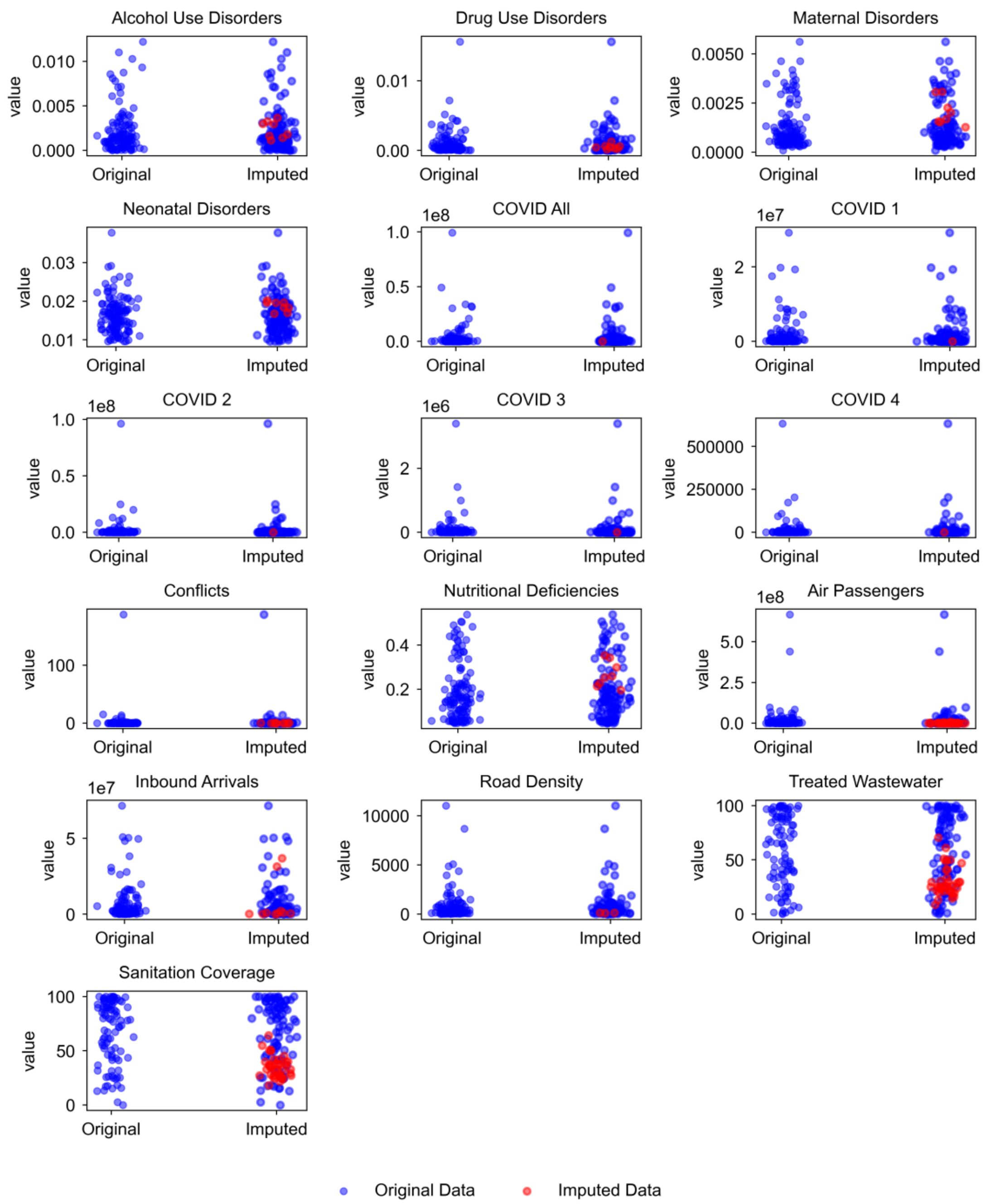

**Fig S5. Kernel density comparison of original and imputed values for variables with missing data (Part1).**

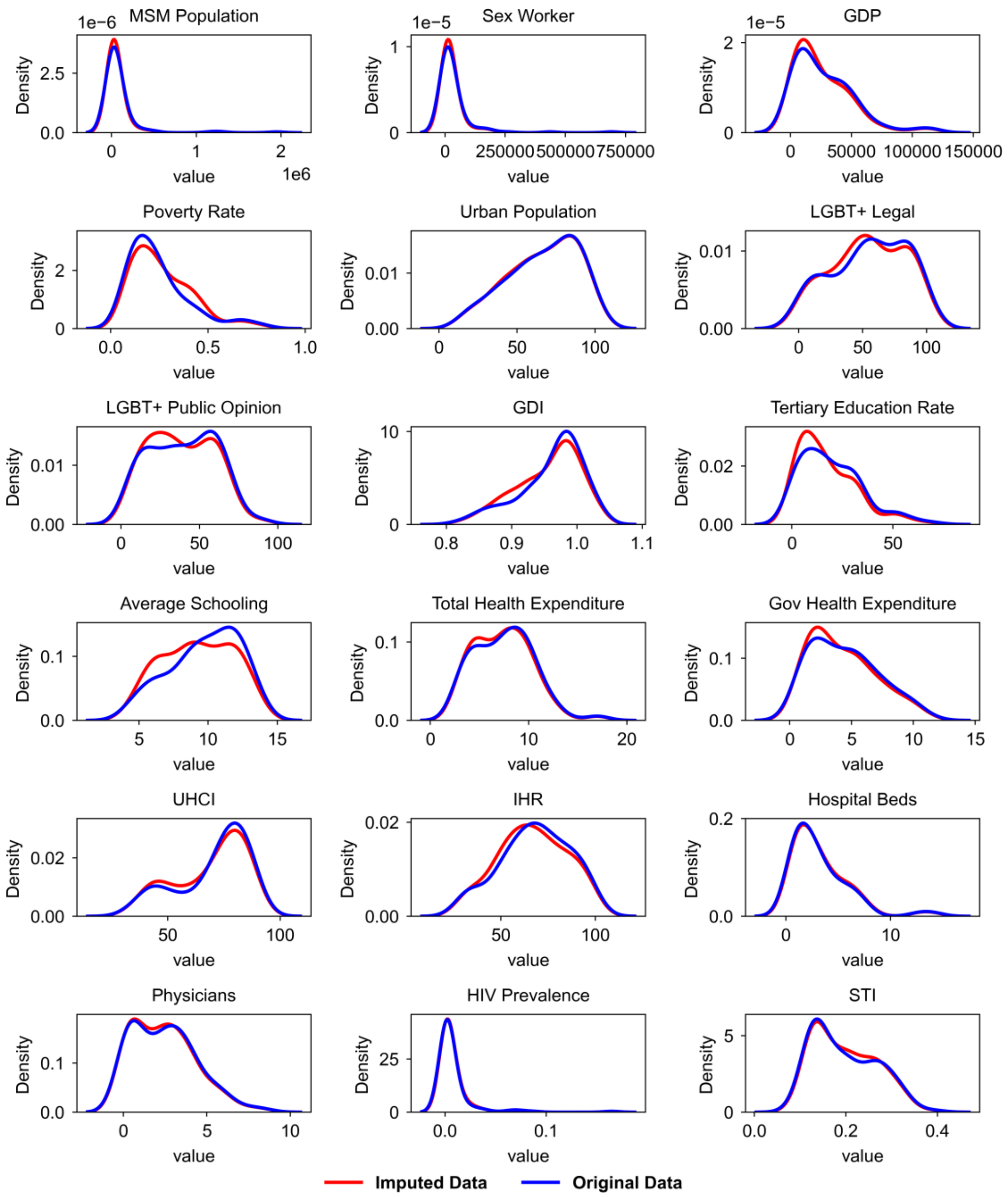

**Fig S6. Kernel density comparison of original and imputed values for variables with missing data (Part2).**

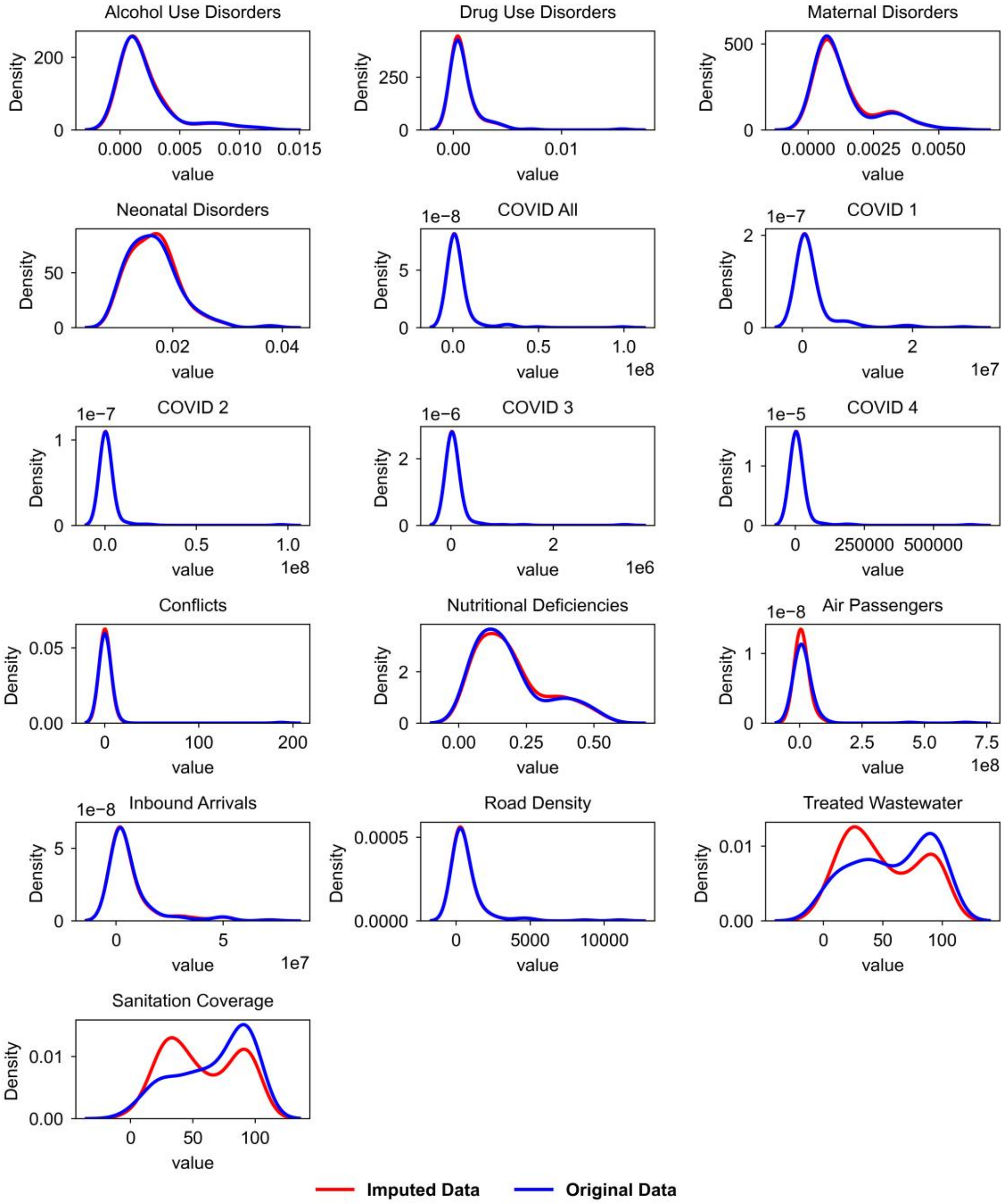

**Fig S7. Cross-validation RMSE for Recursive Feature Elimination with Cross-Validation (RFECV) across different feature selections (a) Period (b)Period 1 (c)Period 2 (d)Period 3 (e) Period 4.**

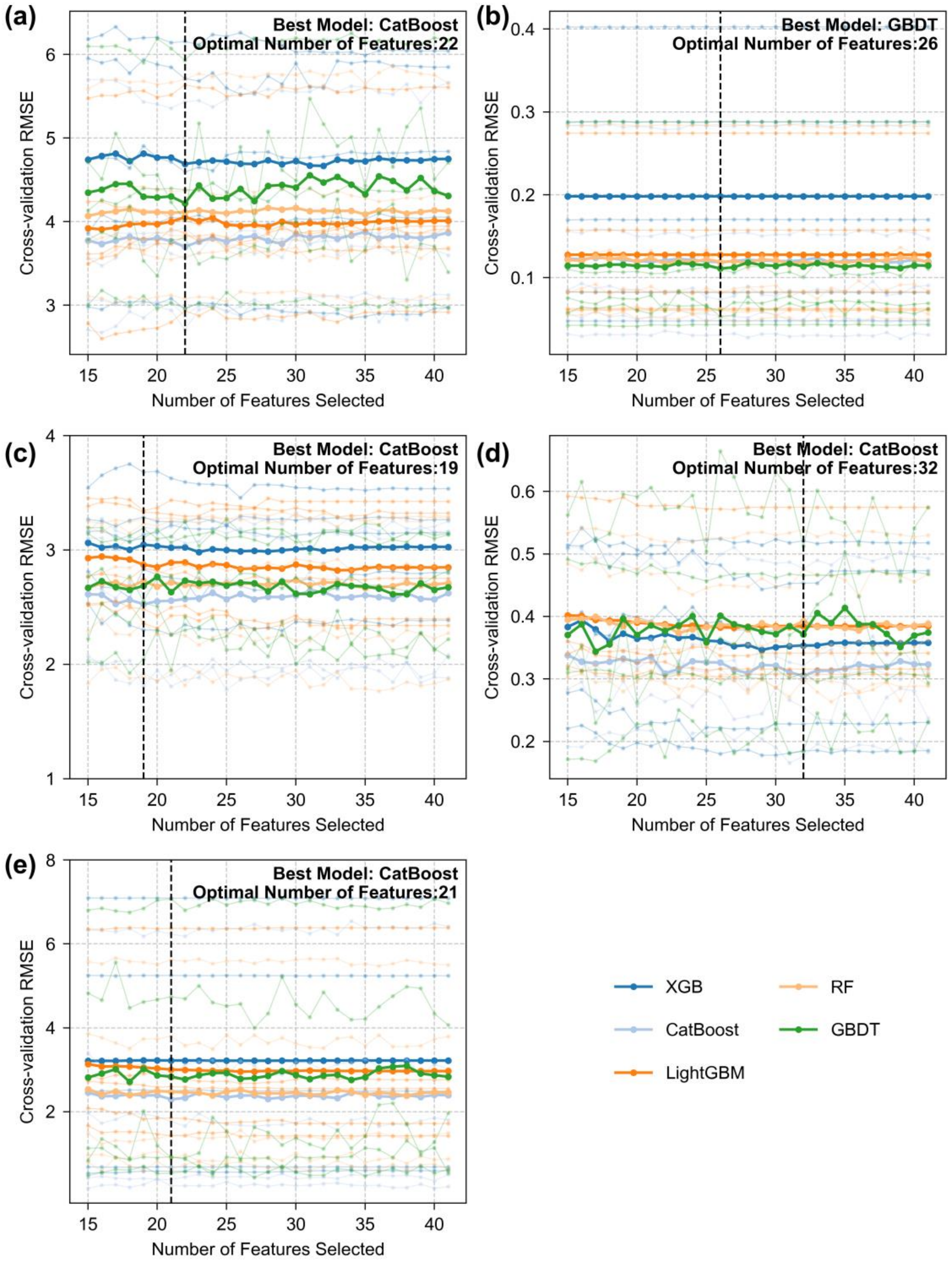

**Fig S8. Multicollinearity filtering based on correlation and feature importance. (a) Period (c)Period1 (e) Period2 Correlation matrix of features selected through RFECV. (b) Period (d)Period1 (f) Period2 Feature importance ranking obtained from regressor model. Features highlighted in red were excluded due to multicollinearity.**

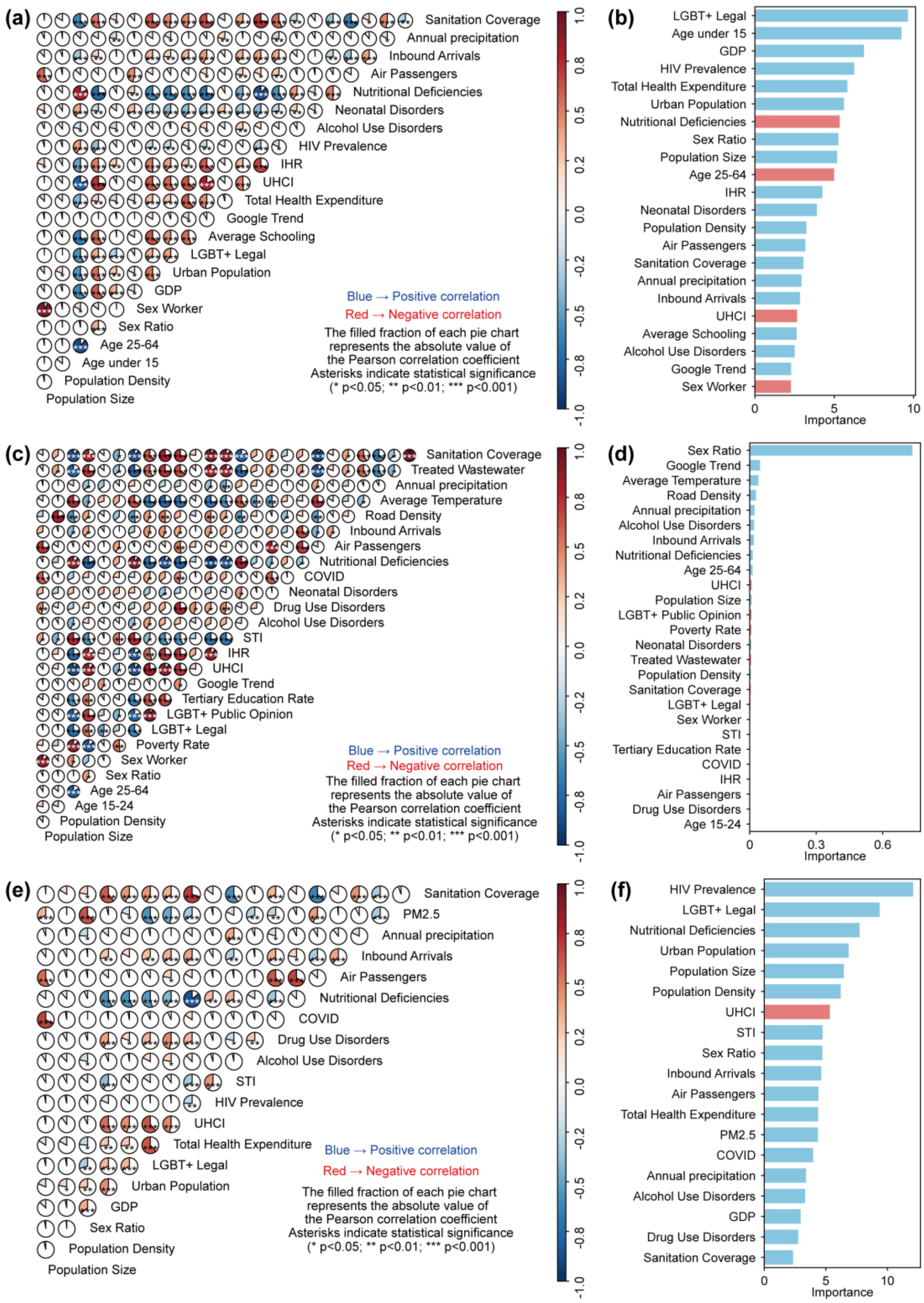

**Fig S9. Multicollinearity filtering based on correlation and feature importance. (a) Period3 (c)Period4 Correlation matrix of features selected through RFECV. (b) Period3 (d)Period4 Feature importance ranking obtained from regressor model. Features highlighted in red were excluded due to multicollinearity.**

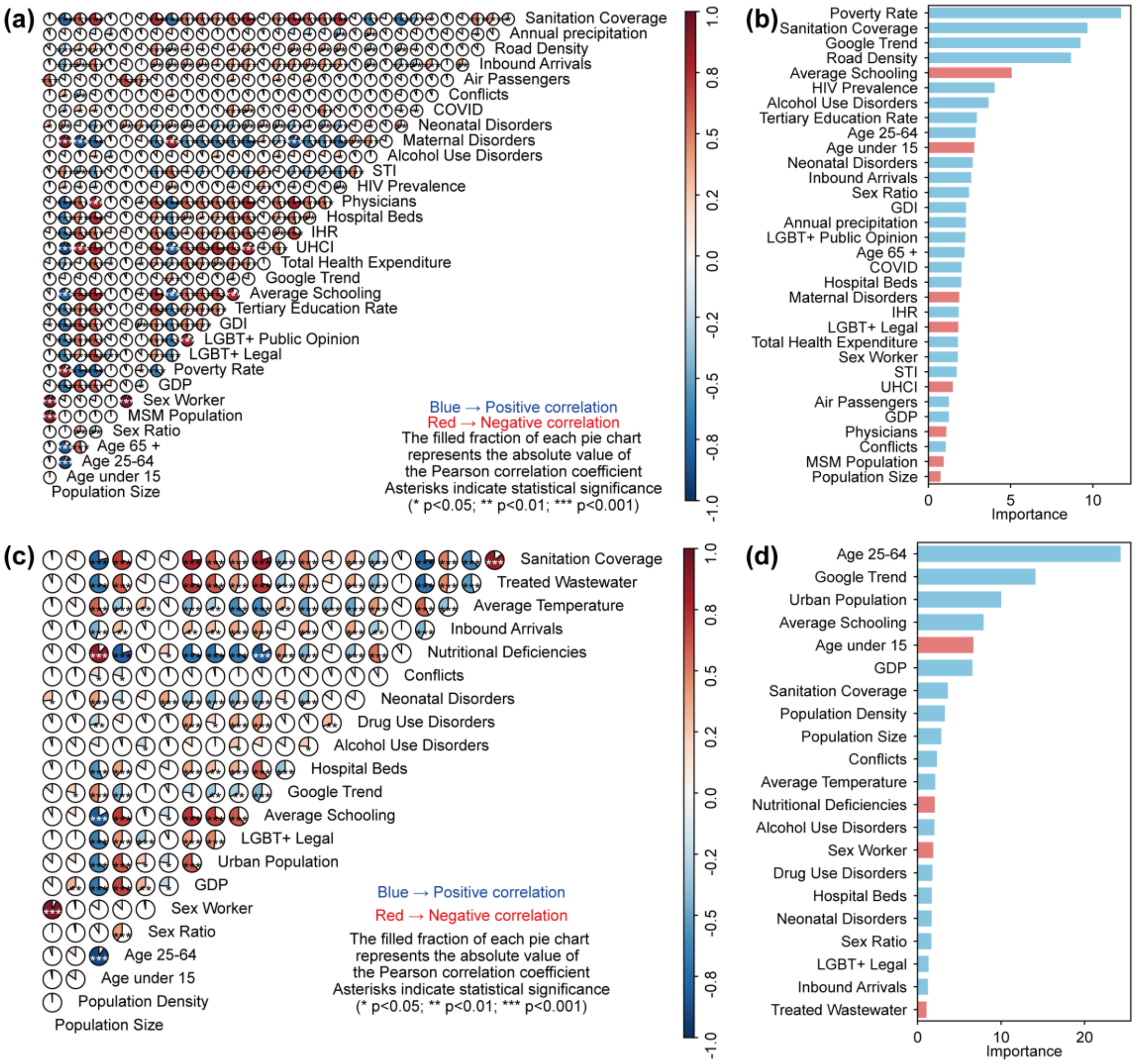

**Fig S10. Spatiotemporal distribution and lineage composition of genotyped mpox counts across epidemic phases. (a) Geographic distribution of MPXV clades (Ia, Ib, IIa, and IIb). Pie charts represent the cumulative proportion of clades in each country, with the radius proportional to the total number of genotyped counts. (b) MPXV clade composition across four epidemic periods (Periods 1-4). Reporting countries are grouped by World Health Organization (WHO) regions. Each pie chart illustrates the lineage distribution for a specific country during a given period, with circle sizes reflecting the corresponding number of genotyped counts.**

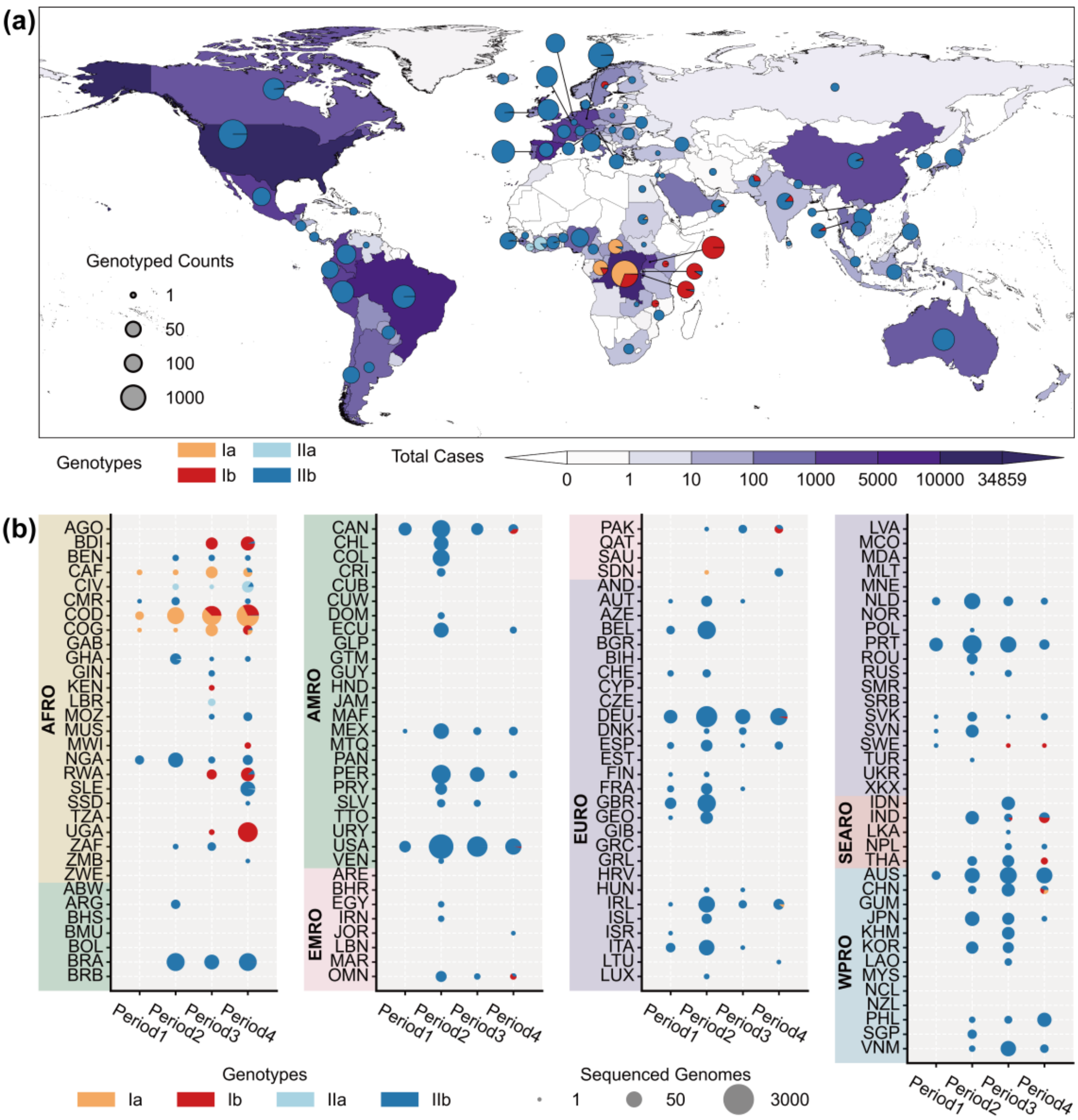

**Fig S11. Regional top five factors of Mpox incidence based on SHAP importance (grouped by WHO regions).**

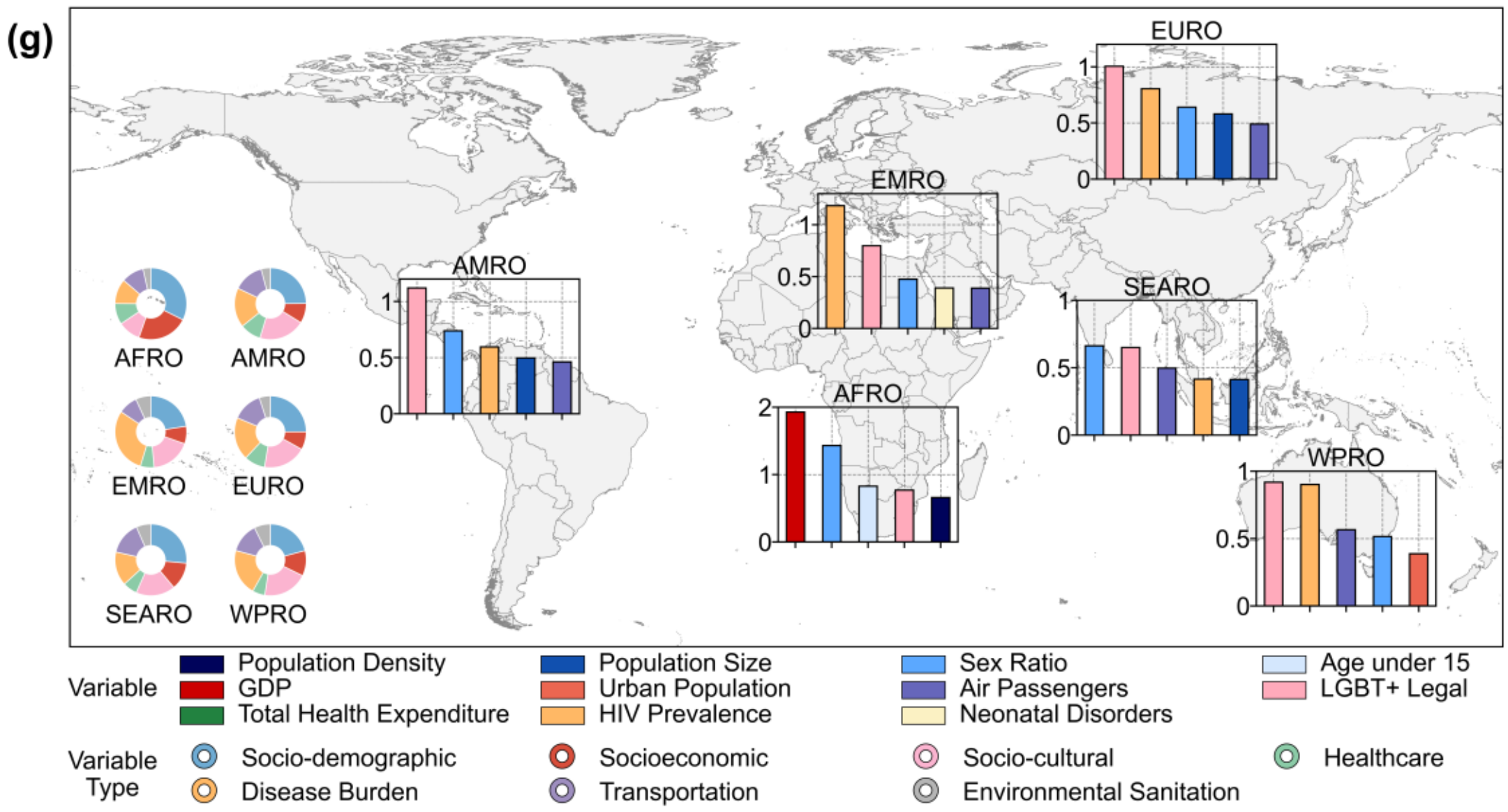

**Fig S12. Scatter plots comparing predicted and observed Mpox incidence rates for training (red) and testing (blue) datasets across four outbreak phases. (a) Period 1, (b) Period 2, (c)Period 3, and (d) Period 4.**

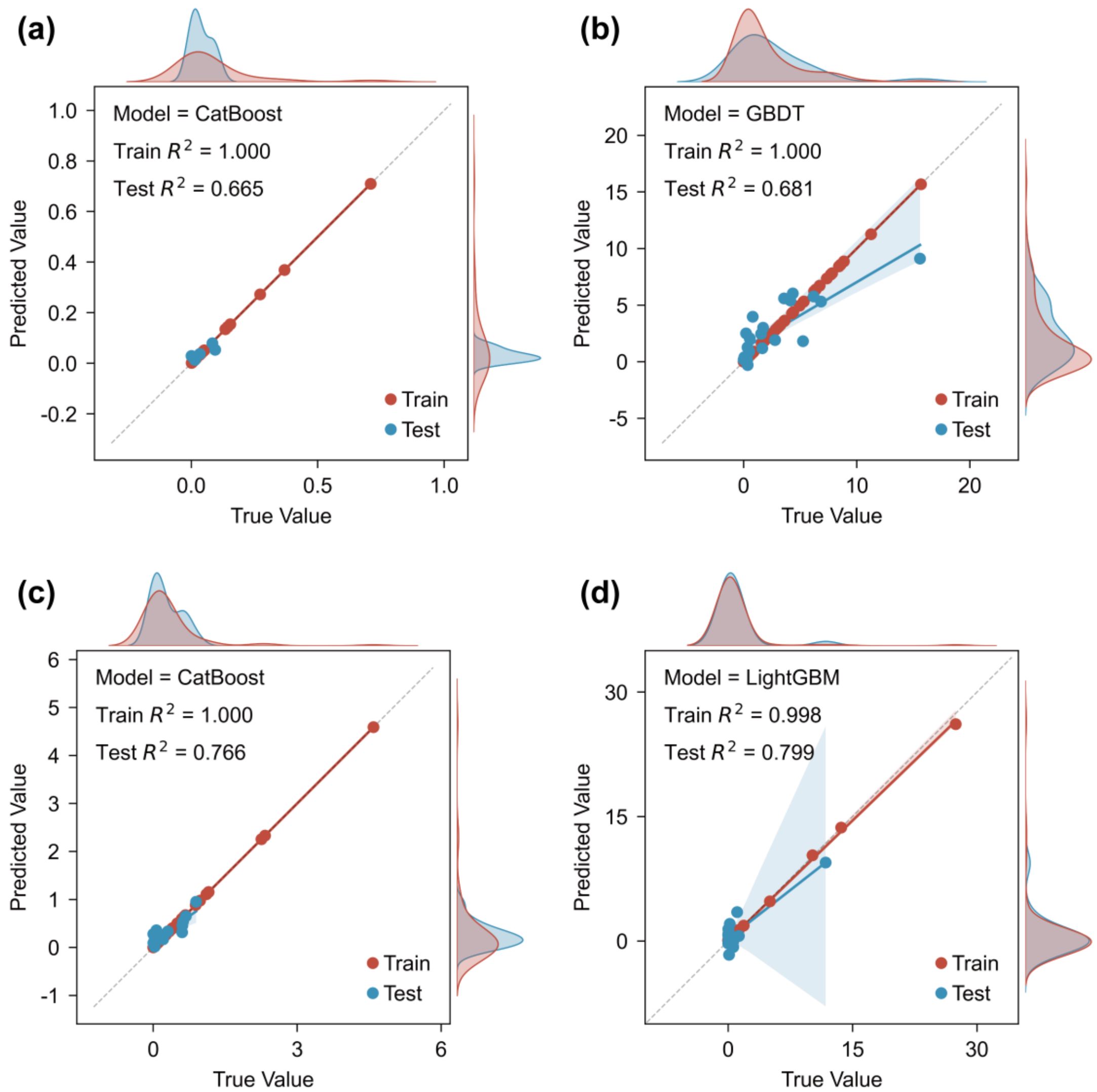

Supplement: Supplementary file 2 [file 12916_2026_5068_MOESM2_ESM.pdf]
