# Supplementary material for: From behavioral to structural correlates: a multi-phase analysis of global reported mpox
Source: BMC Med. 2026 Jul 21;24:484. doi: 10.1186/s12916-026-05068-4 (PMC13563967; doi:10.1186/s12916-026-05068-4)
Supplement: Supplementary file 3 [file 12916_2026_5068_MOESM3_ESM.pdf]

Additional file 3: Texts S1-S3

Text S1. RFECV 1
Text S2. Machine learning models..1
Text S3. Model interpretation. 2

Text S1. RFECV

The method consists of two main components: Recursive Feature Elimination (RFE) and Cross-Validation (CV) [45]. During the RFE phase, the model is trained iteratively on the current feature set, with feature importance assessed in each iteration. The least important features are removed progressively until all features are ranked [46]. In the CV phase, feature subsets are evaluated through cross-validation. The cross-validation procedure identifies the subset size that achieves the highest average score, determining the optimal number of features for the model [47].

Text S2. Machine learning models.

To investigate the relationship between Mpox incidence and its potential drivers, we employed five advanced machine learning models selected for their strong predictive performance and adaptability to structured datasets with mixed feature types: XGBoost, GBDT, LightGBM, Random Forest, and CatBoost.

GBDT (Gradient Boosting Decision Tree) [50] is the foundational boosting model that builds trees sequentially to minimize loss, offering strong interpretability and adaptability to non-linear relationships. XGBoost (eXtreme Gradient Boosting) [51] is an optimized gradient boosting framework known for its high prediction accuracy, efficient computation, and regularization capabilities, making it effective for preventing overfitting in complex datasets. LightGBM (Light Gradient Boosting Machine) [52], designed for large-scale datasets, uses a histogram-based algorithm that significantly improves training speed and reduces memory usage while maintaining high accuracy. Random Forest [53] is a bagging-based ensemble method that constructs multiple decision trees with random feature selection, providing high robustness, reduced variance, and resilience to noisy data. CatBoost (Categorical Boosting) [54] is particularly efficient for datasets containing categorical variables, as it natively handles categorical features without extensive preprocessing and mitigates prediction shift via ordered boosting.

Collectively, these models offer a balance of interpretability, scalability, and predictive power, outperforming many traditional algorithms and thus were well-suited

for the objectives and characteristics of this research.

Text S3. Model interpretation.

SHAP (Shapley Additive exPlanations) [55], grounded in cooperative game theory [56] and local explanations [57], provides a systematic approach to quantify the contribution of each feature to the model's predictions. Specifically, SHAP values help in assessing main effects, which reflect the contribution of individual features, and interaction effects, which capture the joint influence of feature pairs on the prediction [58], offering insights into the underlying factors driving model predictions.

The Shapley value for a feature *i* in the model is computed as follows:

$$\varphi_i = \sum_{S \subseteq N \setminus \{i\}} \frac{|S|!\,(|N| - |S| - 1)!}{|N|!} \left[f_{S\cup\{i\}}\left(x_{S\cup\{i\}}\right) - f_S(x_S) \right] \quad (1)$$

Where *N* represents the set of all features in the model, with |*N*| denoting the total number of features. $N \setminus \{i\}$ is a set of all possible combinations of features excluding i. S is a subset in $N \setminus \{i\}$ and |*S*| is the size of subset S. $f_S(x_S)$is the model prediction using only the features in subset *S*, while $f_{S\cup\{i\}}\left(x_{S\cup\{i\}}\right)$represents the model prediction with features in S plus feature *i*.

This ensures that the contributions of all features add up to the model prediction value for the specific sample [59]:

$$f(x) = \varphi_0(f) + \sum_{i=1}^{n} \varphi_i(f, x) \quad (2)$$

Where$f(x)$represents the predicted value for a specific sample,$\varphi_0(f)$denotes the average value of the target variable of the sample, $\varphi_i(f, x)$represents the contribution of feature $i$to the prediction for observation x.

The interaction values capture the effect of the interaction between two features i and *j* on the model prediction is defined as:

$$\varphi_{i,j} = \sum_{S \subseteq N \setminus \{i,j\}} \frac{|S|!\,(|N| - |S| - 2)!}{2|N-1|!} \left[f_{S\cup\{i,j\}}\left(x_{S\cup\{i,j\}}\right) - f_{S\cup\{i\}}\left(x_{S\cup\{i\}}\right) - f_{S\cup\{j\}}\left(x_{S\cup\{j\}}\right) + f_S(x_S) \right] \quad (3)$$

The main effect of feature i, which reflects the individual contribution of feature i

after accounting for its interactions with other features, is then given by:

$$\varphi_{i,i} = \varphi_i - \sum_{j \neq i} \varphi_{i,j} \tag{4}$$

By decomposing the total contribution into these components, SHAP provides a detailed understanding of both individual feature importance and the synergistic relationships between features in driving model predictions.
